# A Bayesian Two‐Step Multiple Imputation Approach Based on Mixed Models for Missing EMA Data

**DOI:** 10.1002/sim.70325

**Published:** 2025-11-19

**Authors:** Yiheng Wei, Juned Siddique, Bonnie Spring, Donald Hedeker

**Affiliations:** ^1^ Department of Biostatistics, Mailman School of Public Health Columbia University New York New York USA; ^2^ Department of Preventive Medicine, Feinberg School of Medicine Northwestern University Chicago Illinois USA; ^3^ College of Medicine Florida State University Tallahassee Florida USA; ^4^ Department of Public Health Sciences The University of Chicago Chicago Illinois USA

**Keywords:** ecological momentary assessments, informative missing, longitudinal data, mixed model, shared parameter model

## Abstract

Ecological Momentary Assessments (EMA) capture real‐time thoughts and behaviors in natural settings, producing rich longitudinal data for statistical analyses. However, the robustness of these analyses can be compromised by the large amount of missing data in EMA studies. To address this, multiple imputation, a method that replaces missing values with several plausible alternatives, has become increasingly popular. In this article, we introduce a two‐step Bayesian multiple imputation framework which leverages the configuration of mixed models. We adopt and compare: (1) the Random Intercept Linear Mixed model; (2) the Mixed‐effect Location Scale (MELS) model which accounts for subject variance influenced by covariates and random effects; and (3) the Shared Parameter MELS model which additionally links the missing data to the response variable through a random intercept logistic model. Each of these three can be used to complete the posterior distribution within the framework. In the simulation study, we extend this two‐step Bayesian multiple imputation strategy to handle simultaneous missing variables in EMA data and compare the effectiveness of the multiple imputations across the three mixed models. Our analyses highlight the advantages of multiple imputations over single imputations and underscore the importance of selecting an appropriate model for the imputation process. Specifically, modeling within‐subject variance and linking the missingness mechanism to the response will greatly improve the performance in certain scenarios. Furthermore, we applied our techniques to the “Make Better Choices 1 (MBC1)” study, highlighting the distinction, in particular, of imputation results between the Random Intercept Linear Mixed model and the two MELS models in terms of modeling within‐subject variance.

**Abbreviations**
BS: between‐subject
ELPD: expected log predictive density
EMA: ecological momentary assessments
HMC: Hamiltonian Monte Carlo
MAR: missing at random
MBC1: Make Better Choices 1
MCAR: missing completely at random
MCMC: Markov chain Monte Carlo
MELS: mixed‐effects location scale
MNAR: missing not at random
MVPA: moderate‐to‐vigorous physical activity
NUTS: no‐U‐turn sampler
PPC: posterior predictive checking
RILM: random intercept linear mixed
SPMELS: shared parameter mixed effect location scale
WS: within‐subject

## 1 Introduction

### 1.1 Introduction of EMA Study

Ecological Momentary Assessment (EMA) involves repeated sampling of subjects' current behaviors and experiences in their natural environments to assess particular events in subjects lives or assess subjects at periodic intervals. The methodology involves random time sampling, which employs a variety of technologies, ranging from traditional written diaries and telephones to more modern electronic diaries and physiological sensors. The use of EMA allows researchers to compile longitudinal datasets for different subjects, with each subject contributing numerous repeated observations over varying time spans, as designed by the experiment [1]. For example, in the “Make Better Choices 1” (MBC1) study, which aimed to determine if effective interventions could enhance the frequency and consistency of targeted health behaviors, participants were equipped with accelerometers and instructed to use a custom‐designed app. This setup allowed them to self‐monitor their dietary intake and activity levels multiple times throughout the day, providing daily feature data throughout various experimental periods [2, 3].

### 1.2 Missingness for EMA Study and Techniques

In EMA studies, variables can generally be categorized into two types: time‐constant variables and time‐varying variables. Time‐constant variables are typically demographic characteristics collected at baseline, such as age, gender, and education level. Time‐varying variables can be further divided into two subtypes: (1) the target responses that EMA aims to collect repeatedly, such as step counts, heart rate, or emotional states; and (2) time indicators, such as the day of the study or duration within a day. It is common for observations of the first subtype that is, the target responses to be missing due to nonresponse from participants, leading to incomplete data. The missingness in EMA data can exhibit diverse and complex patterns. For example, it may vary across the days of the study: participants tend to be more engaged in the early days, resulting in lower missingness, whereas missingness may increase over time as engagement declines. Additionally, missingness may be related to the unobserved responses themselves; participants experiencing negative outcomes may be less likely to continue participation, resulting in higher missing rates. Finally, missingness may also vary across individuals, reflecting subject‐level heterogeneity. Therefore, it is essential to account for these patterns when imputing missing data in EMA studies to ensure valid inference.

In longitudinal studies, there are typically three types of mechanisms that can generate missing data: the absence of observations is purely random and unrelated to any observed variables, which is referred to as Missing Completely at Random (MCAR); the missing mechanism allows association between the chance of missing and observed data, which is Missing at Random (MAR); or missingness is also determined by unobserved values of the observations, which is Missing not at Random (MNAR) [4]. A common and straightforward method for dealing with missing data is to exclude observations with missing records and work only with complete observations. If the missingness mechanism is MCAR, a complete case analysis can be sensible, although it may well not use all the available information in the data [5]. However, in practice, most missing data scenarios do not adhere to the MCAR assumption. When data are not missing completely at random, conducting a complete case analysis can lead to biased inferences [6].

To address missing data in longitudinal data, various methods have been developed such as pattern mixture models [7, 8], selection models [9], and shared parameter models [10]. In addition to these methods, multiple imputation has emerged as an effective approach, which is to replace missing values by generating multiple different acceptable values representing a distribution of possibilities [11]. Multiple imputation not only retains the advantages of single imputation which, in contrast, generates only one imputed value for each missing observation such as the ability to apply standard complete‐data methods and maintain consistency across users, but also accounts for the uncertainty inherent in the imputation process [12]. However, although multiple imputation has been widely demonstrated to be more effective than single imputation in handling a wide range of missing data problems [13, 14], few studies to date have clearly demonstrated its advantage over single imputation for EMA data sets. Furthermore, for EMA data, although some recent studies have shown the advantage of multiple imputation over other approaches in handling missing data [15], limited research has been conducted comparing the performance of different multiple imputation methods. Therefore, in this article, we aim to compare and assess the potential advantages of newly proposed multiple imputation methods for EMA data over single imputation and to emphasize the choice among different multiple imputation models in this context.

In this article, we employ the two‐step Bayesian approach of multiple imputation, which is well‐established by Schafer [16], that can be derived explicitly through formulas. Specifically, by assuming a posterior distribution for the data y given a parameter set θ, we first derive the posterior distribution of θ based on the observed data and draw m different θ values from this posterior distribution. Then, we draw m imputed values from the posterior distribution of y, conditioning on each of the θ values. A critical component of this two‐step procedure is the choice of an appropriate posterior distribution, which we discuss in the following subsection.

### 1.3 Models for EMA Study

The data collected in EMA studies typically involve the same subjects or entities measured at multiple time points, reflecting a longitudinal structure. One common approach for modeling longitudinal data is the random effects model [17], which is also known as the linear mixed model when applied to continuous normal outcomes. Moreover, to handle missing values of multiple responses or additional covariates, Schafer and Yucel expanded the linear mixed model to create multiple imputations of missing values by a straightforward and effective Markov chain Monte Carlo (MCMC) procedure [18].

In terms of modeling EMA data, Hedeker et al. extended the linear mixed model by incorporating log‐linear models for both the within‐subject (WS) and between‐subject (BS) variances, in addition to the usual modeling of the mean structure [19]. This extension allows for the potential influence of covariates on the means and both sources of variation and permits the inclusion of a random subject effect in the WS variance specification. There have been some recent extensions of this modeling approach to jointly model the longitudinal EMA data and the missingness inherent in the data. Specifically, Cursio et al. proposed a joint model of the intensive longitudinal data and the missingness of the data using time of day and day of week indicator variables [20]. Lin et al. further developed a shared parameter modeling approach that links the primary longitudinal outcome with potentially informative missingness by introducing a random effect on the missing pattern for each subject [21]. Lin also proposed a fully Bayesian estimation approach using the MCMC method. By establishing a connection between the observed data and the potential missingness, Lin successfully demonstrated that parameter estimation is robust under the MNAR missing mechanism, which is likely in real‐world EMA studies. For example, it is intuitive to suspect that for participants with worse behavioral outcomes might be less active in participating in the study, and respond less frequently as a result. Thus, accounting for the possibility of an MNAR mechanism is important when handling missing data in EMA research.

In this article, we will establish the two‐step Bayes approach for multiple imputations based on several mixed models, and compare the imputation performance under different aspects and criteria. By comparing mixed models of increasing complexity, we aim to provide guidance for choosing an appropriate model for imputation of missing EMA data. In terms of the organization of this article, the statistical analysis, which includes the introduction of models, imputation method, and estimation approach, is provided in Section 2. In Section 3, we will analyze the performance of our multiple imputations through simulated EMA data. In Section 4, we will apply our methods to a dataset and analyze the performance. Conclusions and discussion are provided in Section 5.

## 2 Statistical Analysis

### 2.1 Models

Let $y_{ij}$ represent the outcome for subject i at occasion j, and $x_{ij}$ represent the p×1 covariate vector for subject i at occasion j, where i=1,2,…,N and $j=1,2,\ldots ,n_{i}$. Note that $x_{ij}$ can represent both time‐varying and time‐invariant covariates. In this two‐level modeling framework, we are dealing with observations from various subjects, and within each subject, we have observations across different occasions. A basic model for longitudinal data is the Random Intercept Linear Mixed (RILM) model [17]:

(1) $$\begin{array}{ll} & y_{ij}|x_{ij},v_{0,i}∼𝒩\left(\beta_{0}+x_{ij}^{⊤}\beta +v_{0,i},\;\sigma_{\epsilon }^{2}\right) \end{array}$$

(2) $$\begin{array}{ll} & \sigma_{\epsilon }^{2}=e^{\alpha_{0}} \end{array}$$

(3) $$\begin{array}{ll} & v_{0,i}∼𝒩\left(0,\sigma_{v_{0}}^{2}\right), \end{array}$$

where $\beta_{0}$ is the intercept coefficient, β is the p×1 coefficient vector for the covariates, and $v_{0,i}$ is termed the random location effect for subject i, which follows a normal distribution with zero mean and fixed variance $\sigma_{v_{0}}^{2}$. $\sigma_{\epsilon }^{2}$ is the fixed WS variance for $y_{ij}$. To align with the expressions of the subsequent models, we use an exponential expression, $e^{\alpha_{0}}$, to represent the fixed WS variance here.

The importance of modeling the WS variance for EMA data has been previously well‐discussed [19, 22]. To allow covariates to influence the WS variance, we can expand the model by including covariates in the modeling of the WS variance $\sigma_{\epsilon_{ij}}^{2}$. We can also include a random scale effect in the WS variance model, which can be correlated with the random location effect. This model is termed the Mixed‐effects Location Scale (MELS) model [19]:

(4) $$\begin{array}{ll} & y_{ij}|x_{ij},v_{0,i},v_{1,i}∼𝒩\left(\beta_{0}+x_{ij}^{⊤}\beta +v_{0,i},\;\sigma_{\epsilon_{ij}}^{2}\right) \end{array}$$

(5) $$\begin{array}{ll} & \sigma_{\epsilon_{ij}}^{2}=e^{\alpha_{0}+x_{ij}^{⊤}\alpha +v_{1,i}} \end{array}$$

(6) $$\begin{array}{ll} & \left(\begin{array}{c} v_{0,i}\\ v_{1,i} \end{array}\right)∼𝒩\left(\left(\begin{array}{c} 0\\ 0 \end{array}\right),\left(\begin{array}{cc} \sigma_{v_{0}}^{2} & \rho_{v_{0},v_{1}}\sigma_{v_{0}}\sigma_{v_{1}}\\ \rho_{v_{0},v_{1}}\sigma_{v_{0}}\sigma_{v_{1}} & \sigma_{v_{1}}^{2} \end{array}\right)\right). \end{array}$$

Here, $\alpha_{0}$ is the coefficient for the intercept and α is the p×1 vector of coefficients in the model of $y_{ij}$'s conditional variance $\sigma_{\epsilon_{ij}}^{2}$. $v_{1,i}$ is the random scale effect for each subject, which follows a normal distribution with zero mean and a fixed variance $\sigma_{v_{1}}^{2}$. $\rho_{v_{0},v_{1}}$ is the correlation between the random location effect $v_{0,i}$ and random scale effect $v_{1,i}$.

As mentioned, in EMA data, missingness can occur and exhibit diverse patterns under different conditions. For example, missingness can vary across different days of the study, time ranges within a day, and among different subjects. This variability underscores the need for a model that accounts for the probability of missing data using covariates. Similar to the random effects model for the longitudinal continuous responses, we can also model the missingness indicator using covariates and random effects:

(7) $$\begin{array}{l} m_{ij}|t_{ij},\lambda_{i}∼ℬ\left(L\left(\tau_{0}+t_{ij}^{⊤}\tau +\lambda_{i}\right)\right), \end{array}$$

where $m_{ij}$ is the missing indicator for subject i at occasion j. L denotes the logistic function, which follows a Bernoulli distribution ℬ. $\tau_{0}$ is the intercept. $t_{ij}$ denotes the q×1 vector of covariates, and τ is the corresponding q×1 vector of coefficients. We also include a random effect, denoted as $\lambda_{i}$, which we term the random missing effect in this model.

Furthermore, the mean and variance of $y_{ij}$ can also be related to the missingness. For instance, individuals with tight schedules may experience higher stress levels and be less likely to respond to the requests of the EMA study, resulting in missingness related to stress (i.e., one of the EMA variables of interest). Hence, it is reasonable to include the random missing effect in the modeling of both the mean and variance of $y_{ij}$. Continuing in the MELS setting, we employ the Shared Parameter Mixed‐effects Location Scale (SPMELS) model here [21]. This model divides the random location and scale effects into a missing‐decided part and a missing‐orthogonal part:

(8) $$\begin{array}{ll} & v_{0,i}=\gamma \lambda_{i}+\eta_{0,i} \end{array}$$

(9) $$\begin{array}{ll} & v_{1,i}=\delta \lambda_{i}+\eta_{1,i}. \end{array}$$

In Equations (8) and (9), γ and δ are parameters that indicate the degree to which the random missing effect $\lambda_{i}$ influences the random location effect $v_{0,i}$ and the random scale effect $v_{1,i}$, respectively. $\eta_{0,i}$ and $\eta_{1,i}$ are two error terms, with corresponding residual variance $\sigma_{\eta_{0}}^{2}$ and $\sigma_{\eta_{1}}^{2}$, which are orthogonal to $\lambda_{i}$. We term these two parameters the residual random location effect and the residual random scale effect. Since we allow correlation between the random location effect $v_{0,i}$ and random scale effect $v_{1,i}$, there will also exist correlation $\rho_{\eta_{0},\eta_{1}}$ between $\eta_{0,i}$ and $\eta_{1,i}$.

In summary, SPMELS is structured as follows:

(10) $$\begin{array}{ll} & y_{ij}|x_{ij},\eta_{0,i},\eta_{1,i},\lambda_{i}∼𝒩\left(\beta_{0}+x_{ij}^{⊤}\beta +\eta_{0,i}+\gamma \lambda_{i},\sigma_{\epsilon_{ij}}^{2}\right) \end{array}$$

(11) $$\begin{array}{ll} & \sigma_{\epsilon_{ij}}^{2}=e^{\alpha_{0}+x_{ij}^{⊤}\alpha +\eta_{1,i}+\delta \lambda_{i}} \end{array}$$

(12) $$\begin{array}{ll} & m_{ij}|t_{ij},\lambda_{i}∼ℬ\left(L\left(\tau_{0}+t_{ij}^{⊤}\tau +\lambda_{i}\right)\right) \end{array}$$

(13) $$\begin{array}{ll} & \left(\begin{array}{c} \eta_{0,i}\\ \eta_{1,i}\\ \lambda_{i} \end{array}\right)∼𝒩\left(\left(\begin{array}{c} 0\\ 0\\ 0 \end{array}\right),\left(\begin{array}{ccc} \sigma_{\eta_{0}}^{2} & \rho_{\eta_{0},\eta_{1}}\sigma_{\eta_{0}}\sigma_{\eta_{1}} & 0\\ \rho_{\eta_{0},\eta_{1}}\sigma_{\eta_{0}}\sigma_{\eta_{1}} & \sigma_{\eta_{1}}^{2} & 0\\ 0 & 0 & \sigma_{\lambda }^{2} \end{array}\right)\right). \end{array}$$

### 2.2 Two‐Step Multiple Imputation

Imputation will follow a two‐step parametric Bayesian approach [23]. Denote $y_{obs}$ as the observed data and $y_{miss}$ as the missing data, θ is a parameter set for y's distribution. Because

(14) $$\begin{array}{ll} & P\left(y_{miss}|y_{obs}\right)=\int P\left(y_{miss}|y_{obs},\theta \right)P\left(\theta |y_{obs}\right)d\theta , \end{array}$$

an imputation for $y_{miss}$ can be created by first sampling

(15) $$\begin{array}{ll} & \hat{\theta }∼P\left(\theta |y_{obs}\right), \end{array}$$

and then imputing the $y_{miss}$ by sampling

(16) $$\begin{array}{ll} & {ŷ}_{miss}∼P\left(y_{miss}|y_{obs},\hat{\theta }\right). \end{array}$$

By repeating the sampling L times, we can obtain L estimated results of the combination ${\left\{{\hat{\theta }}^{l},{ŷ}_{miss}^{l}\right\}}_{l=1,\ldots ,L}$.

Specifying the parameter set θ and the distribution of y conditional on θ is required before sampling. We will discuss how to estimate the parameter set θ based on the observed data $y_{obs}$, which is $P\left(\theta |y_{obs}\right)$ in step one, in the next subsection “Parameter estimation”.

In this study, we will adopt the RILM, MELS, and SPMELS models described above. The equations and discussion above provides a general overview of the two‐step multiple imputation approach. However, further details for each of the three models can be found in Appendix A.

### 2.3 Parameter Estimation

The methods for estimating the parameters θ differ among these three models. For RILM, many existing packages are available that can yield results efficiently. For example, the R package lme4 employs maximum likelihood estimation [24]. To avoid the challenges of multi‐dimensional integration in maximum likelihood estimation and the computation of the inverse Hessian matrix for MELS and SPMELS, we utilize an MCMC method described by Lin et al. [21]. This method is to sample the Bayesian posterior, $P\left(\theta |y_{obs}\right)$, the distribution of parameters given observed data $y_{obs}$, which can be derived by

(17) $$\begin{array}{ll} & P\left(\theta |y_{obs}\right)\propto L\left(y_{obs}|\theta \right)\pi \left(\theta \right), \end{array}$$

where π(θ) denotes the prior, and $L\left(y_{obs}|\theta \right)$ denotes the likelihood of observed data for a given θ value. The sampling process is conducted in RStan (the R interface to Stan) [25], which generates the posterior samples through the Hamiltonian Monte Carlo (HMC) algorithm and its adaptive variant the no‐U‐turn sampler (NUTS) [26, 27]. For more details of the parameter set θ and likelihood function for each model, please refer to Appendix B.

It should be noted that for estimating the parameters of the RILM, with non‐informative priors, both maximum likelihood and MCMC methods are straightforward to implement and provide near‐identical results. In the simulation study (Section 3), we employ maximum likelihood due to its computational efficiency. However, in the examples section (Section 4), we opt for the MCMC method to compute the Expected Log Predictive Density (ELPD) [28], which is utilized to compare the performance of the three models in that section.

## 3 Simulation Study

We simulated a dataset including two covariates, denoted as ${x_{1}}_{ij}$ and ${x_{2}}_{i}$, along with the response variable $y_{ij}$. ${x_{1}}_{ij}$ and $y_{ij}$ are time‐varying variables that change over subjects and occasions, and ${x_{2}}_{i}$ is a time‐constant continuous variable representing some baseline characteristic.

We adopt the SPMELS model to create the missingness for outcome $y_{ij}$ and time‐varying variable ${x_{1}}_{ij}$. The simulated data set contains 20 subjects, 5 days, and 6 beeps within a day. After each beep, subjects were required to report their real‐time continuous variables, $y_{ij}$ and ${x_{1}}_{ij}$. However, due to non‐response, both the values of $y_{ij}$ and ${x_{1}}_{ij}$ are missing together for some beeps. We assume the missingness tends to increase with study days, and also during the early morning and late night. To realize this, we introduce three variables: $t_{ij}$, ${beep_{1}}_{ij}$, and ${beep_{6}}_{ij}$. $t_{ij}$ takes on integers ranging from 1 to 5, reflecting the different days of data collection. ${beep_{1}}_{ij}$ and ${beep_{6}}_{ij}$ are binary variables that indicate whether the response corresponds to the first beep or the sixth beep, respectively. These beeps occur during the early morning and late night. Additionally, to control the missing ratio to around 20% in each simulation, we adjust the intercept for the missing model for each simulation, which is denoted as $\tau_{0}$. The simulated process can be summarized as the following four steps:

1. Simulate ${x_{2}}_{i}$ by
(18) $$\begin{array}{ll} & {x_{2}}_{i}∼𝒩\left(\mu_{x_{2}},\sigma_{x_{2}}^{2}\right). \end{array}$$
2. Simulate the random missing effect $\lambda_{i}$ and missing indicator $m_{ij}$ by
(19) $$\begin{array}{ll} & \lambda_{i}∼𝒩\left(0,\sigma_{\lambda }^{2}\right) \end{array}$$
(20) $$\begin{array}{ll} & m_{ij}∼ℬ\left(L\left(\tau_{0}+\tau_{1}t_{ij}+\tau_{2}{beep_{1}}_{ij}+\tau_{3}{beep_{6}}_{ij}+\lambda_{i}\right)\right). \end{array}$$
3. Simulate ${x_{1}}_{ij}$ by
(21) $$\begin{array}{ll} & \left(\begin{array}{c} \eta_{0,i}^{\left(1\right)}\\ \eta_{1,i}^{\left(1\right)} \end{array}\right)∼𝒩\left(\left(\begin{array}{c} 0\\ 0 \end{array}\right),\left(\begin{array}{cc} \sigma_{\eta_{0}^{\left(1\right)}}^{2} & \rho_{\eta_{0}^{\left(1\right)},\eta_{1}^{\left(1\right)}}\sigma_{\eta_{0}^{\left(1\right)}}\sigma_{\eta_{1}^{\left(1\right)}}\\ \rho_{\eta_{0}^{\left(1\right)},\eta_{1}^{\left(1\right)}}\sigma_{\eta_{0}^{\left(1\right)}}\sigma_{\eta_{1}^{\left(1\right)}} & \sigma_{\eta_{1}^{\left(1\right)}}^{2} \end{array}\right)\right) \end{array}$$
(22) $$\begin{array}{ll} & {x_{1}}_{ij}|{x_{2}}_{i},\eta_{0,i}^{\left(1\right)},\eta_{1,i}^{\left(1\right)},\lambda_{i}∼𝒩\left(\beta_{0}^{\left(1\right)}+\beta_{2}^{\left(1\right)}{x_{2}}_{i}+\eta_{0,i}^{\left(1\right)}+\gamma^{\left(1\right)}\lambda_{i},{\sigma_{\epsilon_{ij}}^{2}}^{\left(1\right)}\right) \end{array}$$
(23) $$\begin{array}{ll} & {\sigma_{\epsilon_{ij}}^{2}}^{\left(1\right)}=e^{\alpha_{0}^{\left(1\right)}+\alpha_{2}^{\left(1\right)}{x_{2}}_{i}+\eta_{1,i}^{\left(1\right)}+\delta^{\left(1\right)}\lambda_{i}}. \end{array}$$
4. Simulate $y_{ij}$ by
(24) $$\begin{array}{ll} & \left(\begin{array}{c} \eta_{0,i}^{\left(2\right)}\\ \eta_{1,i}^{\left(2\right)} \end{array}\right)∼𝒩\left(\left(\begin{array}{c} 0\\ 0 \end{array}\right),\left(\begin{array}{cc} \sigma_{\eta_{0}^{\left(2\right)}}^{2} & \rho_{\eta_{0}^{\left(2\right)},\eta_{1}^{\left(2\right)}}\sigma_{\eta_{0}^{\left(2\right)}}\sigma_{\eta_{1}^{\left(2\right)}}\\ \rho_{\eta_{0}^{\left(2\right)},\eta_{1}^{\left(2\right)}}\sigma_{\eta_{0}^{\left(2\right)}}\sigma_{\eta_{1}^{\left(2\right)}} & \sigma_{\eta_{1}^{\left(2\right)}}^{2} \end{array}\right)\right) \end{array}$$
(25) $$\begin{array}{ll} & y_{ij}|{x_{1}}_{ij},{x_{2}}_{i},\eta_{0,i}^{\left(2\right)},\eta_{1,i}^{\left(2\right)},\lambda_{i}∼𝒩\left(\beta_{0}^{\left(2\right)}+\beta_{1}^{\left(2\right)}{x_{1}}_{ij}+\beta_{2}^{\left(2\right)}{x_{2}}_{i}+\eta_{0,i}^{\left(2\right)}+\gamma^{\left(2\right)}\lambda_{i},{\sigma_{\epsilon_{ij}}^{2}}^{\left(2\right)}\right) \end{array}$$
(26) $$\begin{array}{ll} & {\sigma_{\epsilon_{ij}}^{2}}^{\left(2\right)}=e^{\alpha_{0}^{\left(2\right)}+\alpha_{1}^{\left(2\right)}{x_{1}}_{ij}+\alpha_{2}^{\left(2\right)}{x_{2}}_{i}+\eta_{1,i}^{\left(2\right)}+\delta^{\left(2\right)}\lambda_{i}}. \end{array}$$

For imputation, we first adopt RILM, MELS, and SPMELS, respectively, to impute ${x_{1}}_{ij}$, using all available covariates (${x_{2}}_{i}$, $t_{ij}$, ${beep_{1}}_{ij}$, and ${beep_{6}}_{ij}$). Then, we also use the three models to impute $y_{ij}$ based on ${x_{2}}_{i}$, $t_{ij}$, ${beep_{1}}_{ij}$, ${beep_{6}}_{ij}$, and the imputed values of ${x_{1}}_{ij}$. For single imputation, we only generate one imputed value for each missing observation. For multiple imputation, we generate 10 separate imputations (L=10) and then take the average of these results to obtain the final imputed value.

We repeat the entire imputation procedure including data generation, modeling, and imputation 100 times for both single and multiple imputation, in order to obtain robust and reliable results. Here we use k to denote the kth repetition. To compare the performance of the three models with different sets of parameters values, we modify some of these values, including $\alpha_{0}^{\left(2\right)}$, $\rho_{\eta_{0}^{\left(2\right)},\eta_{1}^{\left(2\right)}}$, $\gamma^{\left(2\right)}$, $\delta^{\left(2\right)}$, which are used to generate $y_{ij}$. We use bias and coverage rate as the criteria to evaluate the estimates of the three different models. Bias is computed for each parameter as the deviation from the true value, which is $(\sum_{k=1}^{100}({\hat{\theta }}_{k}-\theta ))/100$. Let ${\hat{\theta }}_{k}^{l}$ and ${\hat{\theta }}_{k}^{u}$ represent the lower and upper bounds, respectively, of the estimated 95% confidence interval for the maximum likelihood estimation, or the credible interval for the MCMC estimation approach. The coverage rate is the percentage of the intervals that contain the true value, which is simply $\sum_{k=1}^{100}1_{\left\{{\hat{\theta }}_{k}^{l}<\theta <{\hat{\theta }}_{k}^{u}\right\}}$. To see how the estimation of the three different models will affect the imputation, we then calculate the error of imputation by the average of the squared differences between the real values of $y_{ij}$ and their imputed values ${ŷ}_{ij}$, which is $(\sum_{k=1}^{100}(\frac{\sum_{i=1}^{i=N}\frac{\sum_{j=1}^{j=n_{i}}{\left({ŷ}_{ijk}-y_{ijk}\right)}^{2}}{n_{i}}}{N}))/100$. Here, the result by multiple imputation ${ŷ}_{ijk}$ is calculated by $\frac{1}{L}\sum_{l=1}^{L}{ŷ}_{ijk}^{l}$, where L=10.

### 3.1 Modeling Within‐Variance

Compared to RILM, both MELS and SPMELS allow the WS variance to vary, depending on covariates and a random (subject) scale effect, and allow a correlation between the random location and scale effects. To assess the contributions of introducing covariates and the random scale effect in the imputation process, we vary the values of $\alpha_{0}^{\left(2\right)}$, the intercept in the WS model, and $\rho_{\eta_{1}^{\left(2\right)},\eta_{2}^{\left(2\right)}}$, the correlation between the residual random location and scale effects. A smaller $\alpha_{0}^{\left(2\right)}$ indicates that covariates play a larger role in the WS variance model, while a larger $\rho_{\eta_{1}^{\left(2\right)},\eta_{2}^{\left(2\right)}}$ indicates that the random effects are more important in the WS variance model. Both scenarios highlight the insufficiency of using RILM for modeling.

Assessment of bias and coverage rates for $\alpha_{0}^{\left(2\right)}=0.00$ and $\rho_{\eta_{0}^{\left(2\right)},\eta_{1}^{\left(2\right)}}=-0.20$ is provided in Table C1. It is evident that the estimation obtained through MELS and SPMELS are generally reasonable. The coverage rates for these consistently exceed 90%, with most achieving a coverage rate greater than or equal to 95%. However, for RILM, the estimation of $\beta_{1}^{\left(2\right)}$ falls slightly short at 88%. This discrepancy can be attributed to the fact that RILM, constrained by a fixed WS variance, cannot adequately account for changes in variance across occasions. Consequently, it transfers this variance change into $x_{1_{ij}}$, the sole covariate that changes across occasions. Additionally, RILM displays notably poor performance in estimating $\alpha_{0}^{\left(2\right)}$ with a bias of −0.81 and a coverage rate of only 12%. This arises from the fact that RILM relies solely on this parameter to mimic the variance pattern of $y_{ij}$ and attributes all of the WS variance to $\alpha_{0}^{\left(2\right)}$, leading to a large bias for the estimation of $\alpha_{0}^{\left(2\right)}$.

We then increase the value of $\alpha_{0}^{\left(2\right)}$ from 0.00 to 3.00 while keeping $\rho_{\eta_{0}^{\left(2\right)},\eta_{1}^{\left(2\right)}}$ unchanged. The estimation of $\alpha_{0}^{\left(2\right)}=3.00$ is provided in Table C2. When $\alpha_{0}^{\left(2\right)}$ is small, the covariates and random scale effects exert a significant influence on the WS variance. However, as $\alpha_{0}^{\left(2\right)}$ becomes larger, the intercept value becomes dominant, reducing the model to an approximately RILM model. Calculated by our simulated data sets, when $\alpha_{0}^{\left(2\right)}=3.00$, the intercept term will determine over 95% of the variance of $y_{ij}$. This in turn reduces the efficiency with which covariates and random scale effects are modeled. Comparing the estimate in Table C1 and Table C2, we can see when $\alpha_{0}^{\left(2\right)}$ increases, estimation of the WS variance, like the intercept $\alpha_{0}^{\left(2\right)}$ and the coefficient of subject‐level covariate $\alpha_{2}^{\left(2\right)}$, become unreliable. For $\alpha_{0}^{\left(2\right)}$, its coverage by MELS decreases from 100% to 43%, and its coverage by SPMELS decreases from 100% to 31%. For $\alpha_{2}^{\left(2\right)}$, its coverage of MELS decreases from 100% to 52%, and its coverage of SPMELS decreases from 100% to 43%. This trend can be attributed to the covariate ${x_{2}}_{i}$, which is designed to mimic a subject‐level baseline characteristic. ${x_{2}}_{i}$ remains more consistent compared to ${x_{1}}_{ij}$, as ${x_{1}}_{ij}$ changes, not only across subjects but also across occasions, but ${x_{2}}_{i}$ changes only across subjects. Thus, the ${x_{2}}_{i}$ term sometimes might be conflated with the intercept term. As the intercept of the variance model increases, MELS and SPMELS cannot distinguish the within‐variance of the response caused by the intercept term and the change of ${x_{2}}_{i}$. Thus, they will inaccurately attribute the WS variance caused by ${x_{2}}_{i}$ to the intercept component or vice versa. This results in unreliable estimation of the coefficients of $\alpha_{0}^{\left(2\right)}$ and $\alpha_{2}^{\left(2\right)}$.

This unreliable estimation leads to increased imputation errors by MELS and SPMELS, as shown in Table C3. In Table C3, $\alpha_{0}^{\left(2\right)}$ is varied from 0.00 to 1.00, 2.00, and 3.00. To evaluate the performance of multiple imputation across different models, the table provides the best and worst models for imputing y for each method used to impute $x_{1}$ and calculates the difference ratio between the best and worst models. The results demonstrate that MELS and SPMELS generally outperform RILM; regardless of the value of $\alpha_{0}^{\left(2\right)}$, the best model is consistently MELS or SPMELS, while RILM is invariably the worst. This underscores the advantage of accounting for WS variance in multiple imputation.

Alternatively, as the value of $\alpha_{0}^{\left(2\right)}$ is increased from 0.00 to 3.00, the errors associated with using MELS and SPMELS as the base model tend to approach the errors of using RILM as the base model. For example, in Table C3, when SPMELS is used to impute $x_{1}$, the smallest errors are achieved under the following conditions: using SPMELS to impute y for $\alpha_{0}^{\left(2\right)}=0.00$ and $\alpha_{0}^{\left(2\right)}=1.00$, and using MELS to impute y for $\alpha_{0}^{\left(2\right)}=2.00$ and $\alpha_{0}^{\left(2\right)}=3.00$, as shown in the “Best” column. The corresponding difference ratios of errors are 13.29%, 12.25%, 11.28%, and 10.22%, respectively, indicating a decreasing trend. This decreasing trend is attributed to the unreliable estimation of the coefficients $\alpha_{0}^{\left(2\right)}$ and $\alpha_{2}^{\left(2\right)}$ by MELS and SPMELS when $\alpha_{0}^{\left(2\right)}$ increases, as previously analyzed. Despite this, the performance of MELS and SPMELS is still better than RILM.

Next, we change the association between the random location and scale effects $\rho_{\eta_{0}^{\left(2\right)},\eta_{1}^{\left(2\right)}}$ from a weak negative correlation to a strong negative correlation. Since RILM cannot model this correlation, it causes RILM's estimation of $\beta_{1}^{\left(2\right)}$ to have a larger bias. In Table C4, as the correlation changes from −0.20 to −0.80, the coverage of RILM's estimation of $\beta_{1}^{\left(2\right)}$ decreases from 88% to 81%. When the correlation between $y_{ij}$'s mean and WS variance is small, the across‐occasion change in $y_{ij}$'s mean primarily relies on ${x_{1}}_{ij}$, the sole covariate that changes across occasions. However, as the correlation increases, the level of $y_{ij}$'s WS variance becomes increasingly influential in determining the across‐occasion change in $y_{ij}$'s mean. As RILM can only use ${x_{1}}_{ij}$ to model this across‐occasion change, the estimation of $\beta_{1}^{\left(2\right)}$ becomes biased when the correlation between $y_{ij}$'s conditional mean and WS variance increases.

Table C3 also shows the errors of imputation from $\rho_{\eta_{0}^{\left(2\right)},\eta_{1}^{\left(2\right)}}=-0.20$ to $\rho_{\eta_{0}^{\left(2\right)},\eta_{1}^{\left(2\right)}}=-0.80$. Since RILM cannot model this correlation, we observe a slight increase in the difference ratio. For example, if we use MELS to impute ${x_{1}}_{ij}$ first, the difference ratio of imputing y increases from 13.40% to 14.42%, and if we use SPMELS to impute ${x_{1}}_{ij}$ first, the difference ratio of imputing $y_{ij}$ increases from 13.29% to 14.37%. Both of these examples indicate the insufficiency of using RILM as the base model when the correlation between the random effects is large.

In summary, initiating with RILM to impute the covariate ${x_{1}}_{ij}$ results in significant errors, irrespective of the subsequent model chosen for imputing $y_{ij}$. For imputing $y_{ij}$, when $\alpha_{0}^{\left(2\right)}$ is relatively small, changes in covariates have a noticeable impact on the variance of $y_{ij}$, emphasizing the need for modeling the WS variance. When $\alpha_{0}^{\left(2\right)}$ is quite large and the variance remains approximately constant, using MELS or SPMELS for imputation yields unreliable estimates thus causing the errors to increase. But these errors are still smaller compared to using RILM. In cases where the random location effect and random scale effect exhibit a high correlation, RILM's estimation of the mean model coefficients can become unreliable. Based on these findings, it is generally recommended to use MELS or SPMELS, instead of RILM, as the base model for multiple imputation. These models are more robust across a range of parameter values and data scenarios, making them a more reliable choice for imputing missing data.

### 3.2 Modeling Random Missing Effects

The difference between MELS and SPMELS is that the latter decomposes the random location effect and random scale effect into one missing‐decided part and one missing‐orthogonal part. In this subsection, we explore various combinations of the parameters $\gamma^{\left(2\right)}$ and $\delta^{\left(2\right)}$ associated with the missing‐decided part. Our aim is to evaluate SPMELS's performance and see whether MELS maintains its efficacy. We assign negative values to $\gamma^{\left(2\right)}$, implying that as a subject's level of response y increases, the probability of the observation being missing tends to decrease. Conversely, for $\delta^{\left(2\right)}$, we allocate positive values. This suggests that as a subject's fluctuation in y grows, the probability of the observation being missing tends to rise.

Table C5 shows the estimation with $\gamma^{\left(2\right)}=-1.50$ and $\delta^{\left(2\right)}=0.50$. Table C6 shows the estimation with $\gamma^{\left(2\right)}=-0.50$ and $\delta^{\left(2\right)}=1.00$. As the magnitude of how the missing random effects associate with the mean of y is increased, denoted by the parameter $\gamma^{\left(2\right)}$, from −0.50 to −1.50, by comparing Table C1 and Table C5, SPMELS does not exhibit a clear better performance over MELS. However, for increasing $\delta^{\left(2\right)}$, some of the estimated parameters of the MELS model become unreliable. The coverage rates of $\alpha_{1}^{\left(2\right)}$ is 92% when $\delta^{\left(2\right)}=0.50$ in Table C1, and decreases to 86% when $\delta^{\left(2\right)}=1.00$ in Table C6. Additionally, we observe an increase in the coverage of $\alpha_{0}^{\left(2\right)}$ in RILM. It increases from 12% to 42% when $\delta^{\left(2\right)}$ changes from 0.50 to 1.00. It is because RILM utilizes only $\alpha_{0}^{\left(2\right)}$ to model the entire expression $\alpha_{0}^{\left(2\right)}+\alpha_{1}^{\left(2\right)}{x_{1}}_{ij}+\alpha_{2}^{\left(2\right)}{x_{2}}_{i}+\eta_{1,i}^{\left(2\right)}+\delta^{\left(2\right)}\lambda_{i}$. When the value of $\delta^{\left(2\right)}$ increases, it shifts the value of the entire expression closer to the true value of $\alpha_{0}^{\left(2\right)}$, which is set to 0.00 in our simulation. This “seemingly” better estimation of $\alpha_{0}^{\left(2\right)}$ also makes the errors by the RILM approach those of MELS and SPMELS, causing a decreasing difference ratio when $\delta^{\left(2\right)}$ increases. However, the increase in coverages and decrease in errors by RILM do not necessarily indicate an improvement in its performance.

When examining the estimate of the random location effect ($\sigma_{v_{0}}^{\left(2\right)}$), the random scale effect ($\sigma_{v_{1}}^{\left(2\right)}$), and the correlation between the random location and scale effect ($\rho_{v_{0}^{\left(2\right)},v_{1}^{\left(2\right)}}$), we find that the bias and coverage for the MELS model are acceptable for all changes of $\gamma^{\left(2\right)}$ and $\delta^{\left(2\right)}$. In Tables C1, C5, and C6, it is evident that MELS provides good estimation of the overall variance and correlation of random effects ($\sigma_{v_{0}}^{\left(2\right)}$, $\sigma_{v_{1}}^{\left(2\right)}$, $\rho_{v_{0}^{\left(2\right)},v_{1}^{\left(2\right)}}$). This suggests that while MELS cannot differentiate between the variances of random effects attributable to missing‐decided or missing‐orthogonal components, it still provides good estimation of the overall variance and correlation of random effects. Therefore, as we increase the magnitude of the association between the missing random effects and the mean or variance of y, SPMELS does not exhibit a clear error reduction compared to MELS, as shown in Table C7. Table C7 illustrates the error changes when $\gamma^{\left(2\right)}$ increases from −0.50 to −1.00 and −1.50, and $\delta^{\left(2\right)}$ increases from 0.50 to 0.75 and 1.00. By comparing the errors of MELS and SPMELS, we do not observe a clear superiority of SPMELS over MELS.

In summary, SPMELS exhibits a slight advantage over MELS in terms of improving the accuracy of estimators when the association between the missing data and y is large. When this association is relatively minor, the performance of MELS is comparable to that of SPMELS. However, since both models provide good estimation of the overall variance and correlation of random effects, they do not show clear differences in imputation errors.

## 4 Application to the Make Better Choices 1 Study

### 4.1 Introduction to the Dataset

Make Better Choices 1 (MBC1) was a clinical trial designed to test the optimal manner to maximize simultaneous healthy changes in diet and activity [3, 29]. A total of 204 eligible participants were recruited; two were excluded from this analysis due to missing intervention start and end dates. Eligibility was based on self‐reported engagement in four health risk behaviors: < 5 daily servings of fruits/vegetables, > 8% of daily calories from saturated fat, < 60 min/day of moderate intensity exercise, and > 120 min/day of sedentary leisure screen time. The study had five weeks in total, including a two‐week baseline‐recording phase, a one‐week first‐period intervention (Rx1), and a two‐week second‐period intervention (Rx23). In the MBC1 trial, there were outcomes and intervention components for both diet and activity, but here we will focus on the activity outcomes and intervention components related to activity. For this, participants were randomly assigned to one of two activity‐related intervention conditions: (1) increase moderate‐to‐vigorous physical activity (MVPA) or (2) decrease sedentary leisure time. For Rx1, participants were asked to close one‐half of the gap between their baseline level of the two targeted behaviors and their ultimate daily goal. For Rx23, they were asked to attain and maintain the ultimate goal levels for the two targeted behaviors assigned. All participants were asked to wear an Actigraph accelerometer (model 7164; Actigraph LLC, Pensacola, Florida) throughout the study to self monitor their activity intensities. Time spent sedentary and in MVPA were calculated using the minutes each day. Table C8 presents descriptive statistics on the demographic and clinical variables during the five‐week study.

A valid day was defined as one in which the wear time met or exceeded 10 h [30]. Consequently, some days' physical and sedentary records are defined as missing in the dataset. The overall missing data rate was 31.93%, exhibiting substantial variability across days and subjects. During weekends, the missing rate was smaller (28.73%) compared to weekdays (39.92%). For some subjects, there were no missing records at all, whereas for other subjects, the missing rate was as high as 94.29%. The standard deviation of the missing rate between subjects was 24.49%. Overall, the average missing data rates for the baseline period, Rx1 period, and Rx23 period were 33.68%, 26.31%, and 32.99%, respectively. Within each subject, missing data rates varied significantly across the different periods. For example, the missing data rates for the third subject was 71.43%, 0.00%, and 7.14%, respectively, for the three periods. In extreme cases, all of the physical and sedentary minutes were missing for one intervention period for some subjects. During the baseline period, all data were missing for 18 subjects, and similarly, all data were missing for 18 subjects during the Rx1 period. Note that these were not necessarily the same individuals. During the Rx23 period, all data were missing for 9 subjects. This variability in missingness highlights the potential importance of modeling the missing data.

### 4.2 Models and Parameter Estimates

In the dataset, the two activity‐related variables, sedentary minutes and MVPA, were missing simultaneously. In the analysis, we treated MVPA as the target variable, and sedentary minutes was imputed before MVPA. Since both of these variables were right‐skewed, a square root transformation was applied to both to approximate a normal distribution. Since the covariate age had a large range, it was divided by 10 to represent changes for a decile of age.

Gender, age, and condition were included as covariates in the regression models. For $condition_{i}$, a value of 0 represents the intervention condition “Increase MVPA” while a value of 1 corresponds to “Decrease sedentary behavior”. Additionally, three binary variables were created: $Rx1_{ij}$, $Rx23_{ij}$, and $weekend_{ij}$. $Rx1_{ij}$ indicates whether the day falls during the Rx1 intervention period, $Rx23_{ij}$ indicates whether the day falls during the Rx23 intervention period, and $weekend_{ij}$ indicates whether the day falls on a weekend. Preliminary analyses of the dataset suggested an interaction among treatment condition, study period, and weekend/weekday [31]. Therefore, interaction terms, including $Rx1_{ij}*condition_{i}$, $Rx23_{ij}*condition_{i}$, $Rx1_{ij}*weekend_{ij}$, $Rx23_{ij}*weekend_{ij}$, $Rx1_{ij}*condition_{i}*weekend_{ij}$, and $Rx23_{ij}*condition_{i}*weekend_{ij}$, were included in the models. The results are presented in Tables C9 and C10.

In Table C9, the regression using sedentary minutes as the response, we observe several significant estimates for the regression coefficients (β). For instance, the estimate of $\beta_{gender}$ is −0.99 for SPMELS, which indicates that females (gender = 1) have, on average, 0.99 fewer (square root) sedentary minutes each day compared to males. Additionally, the significance of $\beta_{Rx23}$, $\beta_{weekend}$, and $\beta_{Rx1*weekend}$ suggests that sedentary minutes significantly change across different intervention periods and time.

For the significant estimates of WS variance, the α parameters, we see that $\alpha_{condition}$ is 0.23 by MELS and 0.24 by SPMELS, indicating that during the “Decrease sedentary minutes” condition, subjects experience a larger variance in sedentary minutes. Additionally, $\alpha_{weekend}$ is 0.18 by both models, suggesting that during weekends, subjects have a larger variance in sedentary minutes.

Turning to the missingness submodel of SPMELS, in terms of the τ parameters we can see the missing ratio is significantly larger during weekends compared to weekdays, and significantly smaller during the Rx1 intervention period compared to baseline. Both estimates of γ and δ are significant. Subjects with lower levels of sedentary minutes or with greater WS variance in sedentary minutes are more likely to have more missing data. In terms of variance explained, missingness accounts for $\frac{{\left(-0.46*1.39\right)}^{2}}{{\left(-0.46*1.39\right)}^{2}+{\left(1.66\right)}^{2}}*100\%\approx 12.91\%$ of the variance of the random location effect and about $\frac{{\left(0.07*1.39\right)}^{2}}{{\left(0.07*1.39\right)}^{2}+{\left(0.38\right)}^{2}}*100\%\approx 6.15\%$ of the variance of the random scale effect.

Table C10 lists the estimates for (the square‐rooted) MVPA as the response variable. The model includes the same covariates used in the regression for sedentary minutes, with the addition of sedentary minutes as a covariate in the regression for MVPA. First, we observe that both the estimates of $\beta_{sedentary\;minutes}$ and $\alpha_{sedentary\;minutes}$ are significant. Take SPMELS as an example. The estimate of $\beta_{sedentary\;minutes}$ is −0.08, indicating that subjects with one more unit of square‐rooted sedentary minutes each day have 0.08 units less square‐rooted MVPA each day. The estimate of $\alpha_{sedentary\;minutes}$ is −0.02, indicating that subjects with one more unit of square‐rooted sedentary minutes each day exhibit 0.02 units less variance in the square‐rooted MVPA each day.

The significance of other estimates shows the mean and variance of MVPA changes based on demographic features, condition, intervention period, and time. Here, we highlight two significant results for α that will be used in the following subsection. The first is $\alpha_{age}$, which is estimated as −0.11 in both models, indicating that older subjects have lower WS variance in their MVPA. The second is $\alpha_{Rx1}$, estimated as −0.20 in both models, indicating that subjects have lower MVPA WS variance during the Rx1 intervention period compared to baseline.

For MVPA's modeling of missingness, both estimates of γ and δ are nonsignificant. Missingness accounts for $\frac{{\left(-0.03*1.40\right)}^{2}}{{\left(-0.03*1.40\right)}^{2}+{\left(1.27\right)}^{2}}*100\%\approx 0.11\%$ of the variance of the random location effect and about $\frac{{\left(-0.02*1.40\right)}^{2}}{{\left(-0.02*1.40\right)}^{2}+{\left(0.44\right)}^{2}}*100\%\approx 0.40\%$ of the variance of the random scale effect. Since these values are quite small, the missingness doesn't show obvious association with the mean and WS variance of MVPA.

Expected Log Predictive Density (ELPD) values were computed for each of the three models [28], which are listed towards the bottom of Table C9 and Table C10. ELPD is calculated through the leave‐one‐out cross‐validation approach, and a higher ELPD indicates better predictive performance. In Table C9, the ELPD of RILM is −108,90.93, much smaller than −10767.78 for MELS and −107,66.08 for SPMELS. In Table C10, the ELPD of RILM is −9688.50, which is also smaller than −9480.24 for MELS and −9480.31 for SPMELS. These suggest that MELS and SPMELS have better fit than RILM. Additionally, the ELPD values for both MELS and SPMELS are comparable, suggesting that these two models exhibit similar performance.

### 4.3 Imputation

After fitting each of the three models for MVPA and sedentary behavior, we then conducted multiple imputation. The imputation results will be shown and described in this subsection.

**FIGURE 1:**
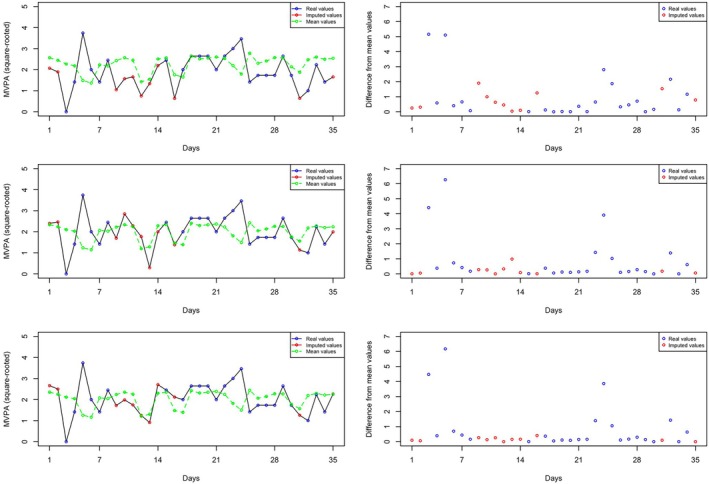
Plots depicting observed and imputed MVPA values across three models for the 75th participant: Initially, SPMELS is used to impute sedentary minutes, followed by the use of RILM, MELS, and SPMELS in rows 1, 2, and 3, respectively, for imputing MVPA.

To illustrate the differences in the imputation results produced by the three different models, we present the plots in Figure 1 which pertains to the 75th participant, whose age is 58, considerably higher than the average age of 32.81. In the plots for the first column, red points represent imputed values, while blue points represent observed values. Additionally, green points are included to show the predicted subject‐level mean for the 75th participant. As shown in Table C10, the estimate of $\alpha_{age}$ is −0.11, and its 95% credible interval does not include zero. This suggests that participants with higher ages have smaller WS variance. As a result, in the plots for the first column, the difference between the red points and the green points is smaller for the MELS and SPMELS models compared to RILM, since RILM does not model WS variance.

To make this difference clearer, the plots in the second column display the squared difference between the observed or imputed values and the mean values. A noticeable difference can be observed in the RILM plot compared to MELS and SPMELS, where the red points' deviations from zero are more pronounced. This indicates that utilizing MELS (the second row) or SPMELS (the third row) to impute MVPA recognizes the smaller WS variance for the 75th participant, while RILM (the first row) does not.

**FIGURE 2:**
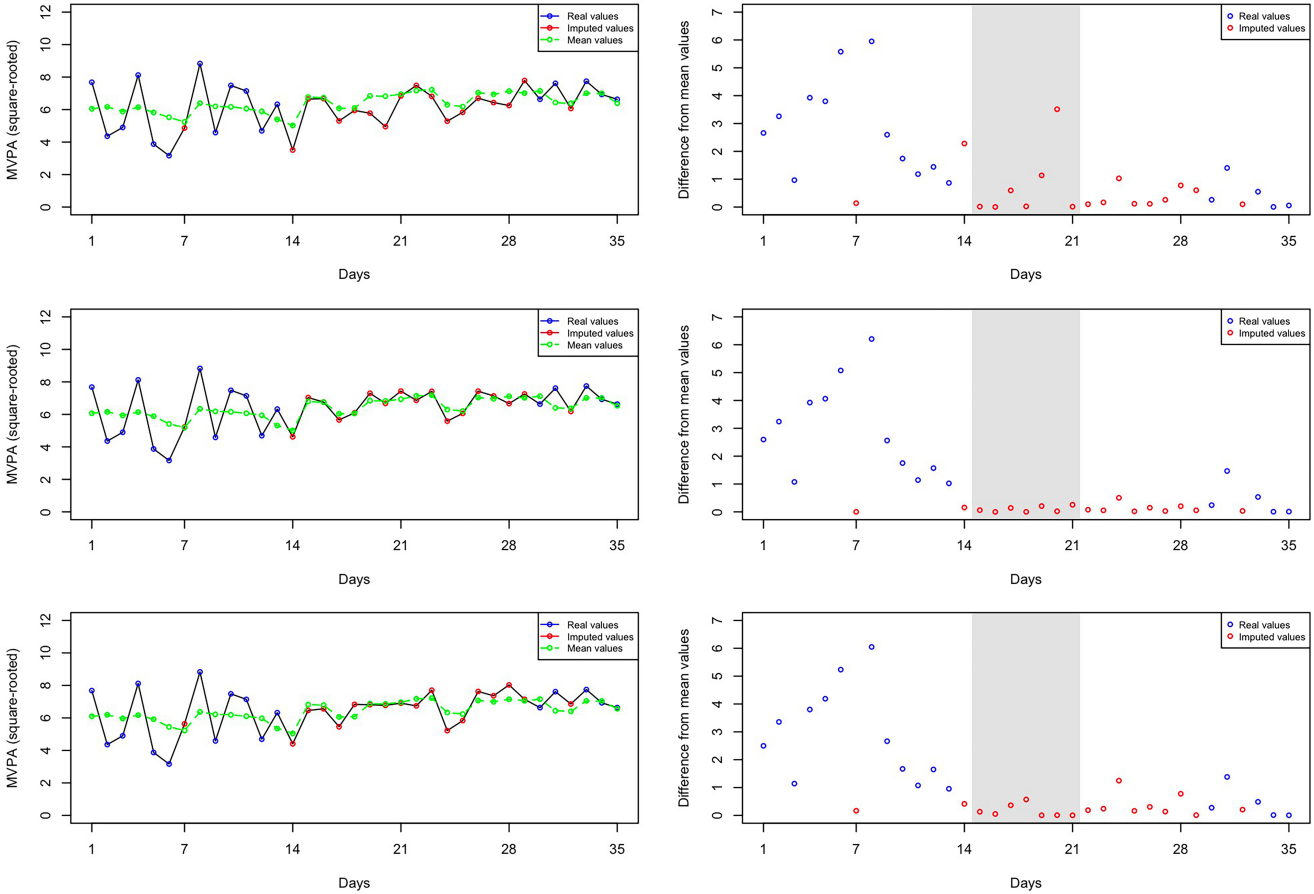
Plots depicting observed and imputed MVPA values across three models for the 19th participant: Initially, SPMELS is used to impute sedentary minutes, followed by the use of RILM, MELS, and SPMELS in rows 1, 2, and 3, respectively, for imputing MVPA.

Another example concerns the effect of the treatment variable Rx1. From Table C10, we observe that the estimate for $\alpha_{Rx1}$ is −0.20, and its 95% credible interval does not include zero. This indicates that compared to the baseline, participants exhibit smaller WS variance during the Rx1 intervention period. Figure 2 shows the imputed values for the 19th participant, highlighting values during the Rx1 intervention period in grey. When using either the MELS or SPMELS model for MVPA, we can capture the smaller WS variance during Rx1. As a result, the differences (from mean values) in the plots of the second and third rows are much smaller compared to RILM (first row) which fails to capture the change in WS variance.

Posterior Predictive Checking (PPC) is a diagnostic approach for imputation models when the true values of missing data are unknown [32]. It assesses whether the analysis results using the completed data conform to those that might have been expected in the absence of missing data. The completed data, denoted as $y_{com}=\left(y_{obs},y_{miss}\right)$, comprises the observed data and the missing data whose values are not known. The replicate of the complete expected dataset is denoted as $y_{com}^{rep}$. PPC assesses the posterior distribution of the completed‐data discrepancy, which is the difference between a function of the completed data $y_{com}$ and their replicates $y_{com}^{rep}$ under the imputation model, expressed as $Q\left(y_{com}^{rep}\right)-Q\left(y_{obs},y_{miss}\right)$, for some analytic interest Q. We calculate the posterior predictive *p*‐value for the imputation model as

(27) $$\begin{array}{ll} p_{com} & =P\left(Q\left(y_{com}^{rep}\right)⩾Q\left(y_{obs},y_{miss}\right)|y_{obs}\right) \end{array}$$

(28) $$\begin{array}{ll} & =\int \int I_{Q\left(y_{com}^{rep}\right)⩾Q\left(y_{obs},y_{miss}\right)}f\left(y_{com}^{rep},y_{miss}|y_{obs}\right)dy_{com}^{rep}dy_{miss}, \end{array}$$

where the *p*‐value $p_{com}$ accounts for the uncertainty about the parameter set θ in evaluating the similarity between $y_{com}$ and $y_{com}^{rep}$. An extreme *p*‐value (close to 0 or 1) suggests that the discrepancy between $y_{com}$ and $y_{com}^{rep}$ may not be easily explained by chance, casting doubt on the model fit.

The *p*‐value $p_{com}$ can be estimated by simulation. For l=1,…,L, we draw $\theta^{l}$ from $f\left(\theta |y_{obs}\right)$. We impute $y_{miss}^{l}$ from $f\left(y_{miss}|\theta =\theta^{l},y_{obs}\right)$, and simulate $y_{com}^{rep,l}$ from $f\left(y_{com}^{rep}|\theta =\theta^{l}\right)$ for each l=1,…,L. We then estimate the *p*‐value by the proportion of the L draws for which $Q\left(y_{com}^{rep,l}\right)⩾Q\left(y_{obs},y_{miss}^{l}\right)$.

In this study, we conducted 1000 simulations (L=1000) for each participant, using the WS variance as the analytic statistic, denoted as Q. The observed data and multiple imputation results for sedentary minutes from the SPMELS model are treated as $y_{obs}$. For each subject, we calculate the posterior predictive check (PPC) p‐value, denoted as $p_{com}$.

**FIGURE 3:**
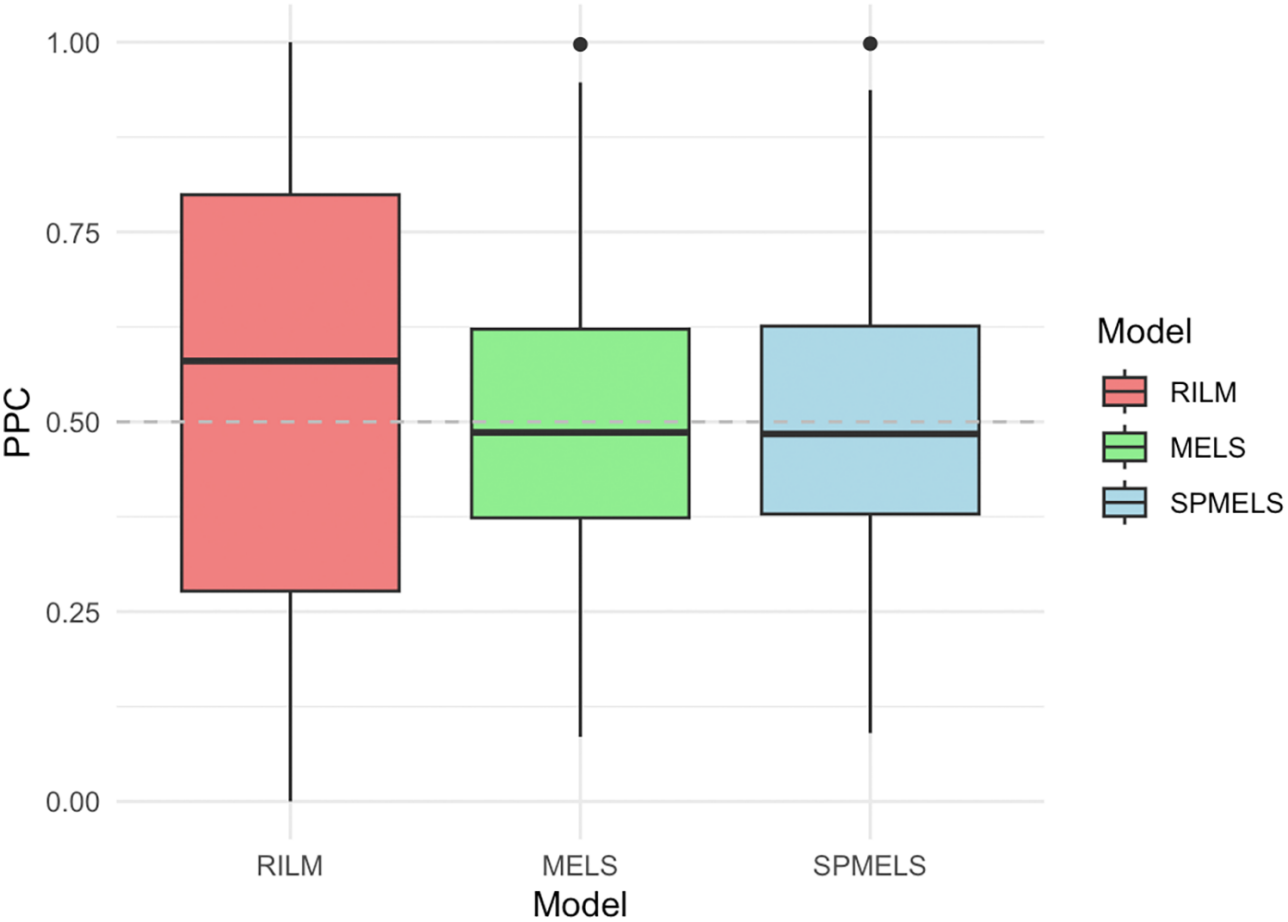
Boxplots of PPC *p*‐values for three models.

Figure 3 presents the boxplot of PPC values across all subjects and for the three models. Compared to MELS and SPMELS, we see that the RILM model (in red) has a median PPC value closer to 1 and exhibits a wider spread in its distribution. In the context of posterior predictive checks, if a model's PPC value is close to 0 or 1 compared to the others, the model may not fit the data well. The relatively high median and broad distribution of PPC values for the RILM model raise concerns about its adequacy due to the omission of WS variance. On the other hand, the narrower and more centered distributions to 0.5 for the MELS and SPMELS models suggest that these models better capture WS variability, leading to a closer alignment with the observed data and a more satisfactory fit.

**FIGURE 4:**
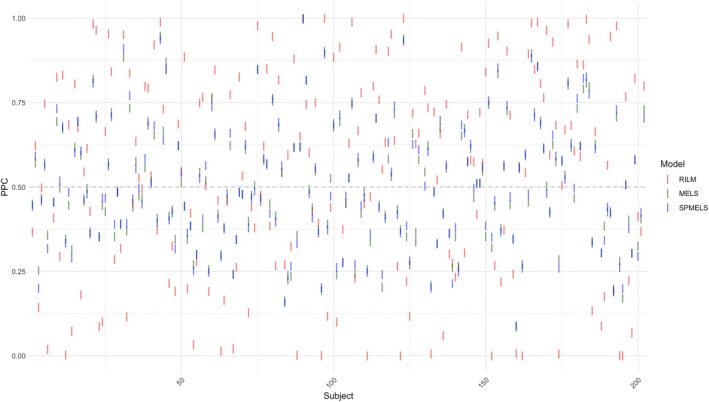
PPC *p*‐values for three models across each subject.

Figure 4 displays the PPC values for the three models across subjects. The x‐axis represents subject IDs from 1 to 202, with the 7th, 129th, and 187th subjects excluded due to non‐missing data. The y‐axis indicates the PPC values, where RILM values are shown in red, MELS in green, and SPMELS in blue. The plot reveals that most of the PPC values for RILM (in red) are distributed closer to 0 or 1 than those for MELS and SPMELS, suggesting that the RILM model may not fit the data. From the figure, we see that many of the PPCs by MELS and SPMELS are overlapping. Thus, MELS and SPMELS generally have similar PPC distributions, indicating comparable model fits. This figure further indicates the importance of accounting for WS variance in modeling to achieve a better fit.

In general, the analysis presented above serves as an illustrative example of the practical application of the proposed multiple imputation methods to a real dataset. It highlights the importance of modeling the WS variance during imputation.

## 5 Discussion

In this article, we adopt a two‐step Bayesian approach for multiple imputation: by assuming a posterior distribution for the data y given a parameter set θ, we first derive the posterior distribution of θ based on the observed data and draw m different θ values from this posterior distribution. We then draw m imputed values from the posterior distribution of y, conditioning on each of the θ values. In this two‐step approach, choosing an appropriate posterior distribution for modeling is important. We employed three distinct models, namely, the Random Intercept Linear Mixed (RILM) model, the Mixed‐effects Location Scale (MELS) model, and the Shared Parameter Mixed Effect Location Scale (SPMELS) model to complete the posterior distribution within this framework. The MELS model extends the RILM by accommodating changes in the within‐subject variance based on covariates and random effects, and the SPMELS model further builds upon the MELS framework by incorporating a random intercept logistic model for intermittent missing data, establishing a link between missing data and the response variable. For model estimation in both MELS and SPMELS, we employ an MCMC approach to alleviate the computational challenges associated with multidimensional numerical integration in maximum likelihood estimation.

Simulation results under the SPMELS model's settings reveal a clear advantage of multiple imputation over single imputation. Of the models employed for multiple imputation, both MELS and SPMELS outperform the RILM, particularly when covariates and random effects exert a moderate to large influence on the WS variance of the response variable. It is worth noting that the performance of MELS and SPMELS, while generally similar, may diverge when the extent of missing data is largely related to observations. Within the SPMELS model, two parameters, γ and δ, are set to quantify the relationship between missing data and the subject's mean and variance, respectively. A large absolute value of γ indicates a significant association between missing data and the subject's mean, and a large absolute value of δ signifies a strong connection between missing data and the within‐subject variance. The simulation suggests that under a large δ value, MELS may lead to less accurate parameter estimation compared to SPMELS, making SPMELS the preferred method.

Following this, we employed our multiple imputation techniques in a case study: the Make Better Choices 1 (MBC1) study. In this case, we observed distinct imputation outcomes using RILM with MELS and SPMELS, primarily attributable to RILM's absence of modeling the WS variance. Additionally, by comparing the PPC *p*‐values of the three models, inadequate model fit of RILM was observed. Therefore, utilizing MELS and SPMELS for modeling WS variance is crucial in multiple imputation.

In conclusion, three key insights can be drawn from these results:

1. Incorporating WS variability into the model improves imputation performance greatly, indicating that a simple RILM model is insufficient;
2. When the goal is to understand the extent to which the response is associated with missingness, fitting the SPMELS model is beneficial;
3. If the primary focus is on reducing imputation error rather than quantifying the association between the response and missingness, the MELS model may be sufficient in some scenarios.

In our model, certain assumptions are made based on common characteristics of EMA data. However, in less typical scenarios, these assumptions may not hold, and more tailored, case‐specific models can be developed in future work. Here are some examples:

1. **Missing of time‐invariant variables:** We assume that time‐invariant variables such as age, gender, or education level are fully observed and missingness only occurs for time‐varying variables. While missingness in the time‐invariant variables is perhaps less common in EMA studies, it can occur. Future work could extend the current model to account for missingness in both time‐varying and time‐invariant covariates.
2. **Homogeneous covariate effects:** Our model assumes that the covariates have the same effect across individuals and includes only a random intercept to account for between‐subject variability. However, effects of time‐varying predictors may differ between individuals. Extending the model to include random slopes would allow for individual‐specific effects and potentially improve imputation performance.
3. **Explicit modeling of WS autocorrelation:** Our model does not explicitly account for autocorrelation in the residuals $\epsilon_{ij}$ for the repeated measures of the same subject, which can be present in EMA data. While we include subject‐specific random effects for WS variance that induce some degree of autocorrelation, this approach is relatively inflexible and does not directly model the temporal dependence between observations. A more direct and flexible approach would be to incorporate person‐specific autocorrelation parameters into the model, as demonstrated in Nestler (2022, SIM) and Nestler (2024, MBR) [33, 34].
4. **Normality assumption:** While our approach concentrated on the conventional and widely studied normal distribution, within the same Bayesian framework, our multiple imputation method might be extended to accommodate other types of outcomes, including binary and Poisson outcomes. These extensions can significantly broaden the applicability of our method to a wider range of research scenarios, where the nature of the data may not conform to a normal distribution.
5. **Sequential imputation for multivariate missingness:** Due to the nature of EMA studies, many EMA data sets exhibit simultaneous missingness across multiple variables. In this study, we employ a step‐by‐step technique to impute these multiple missing variables sequentially. However, such a step‐by‐step training and imputation process can be time‐consuming. It would be beneficial to obtain the joint distribution of all the missing variables and impute them simultaneously. One possible method is to use the Gibbs sampling technique, which iteratively samples from the full conditional distributions for each variable. Future work could entail further exploration in this area.

Overall, as an initial examination of using the MELS model for imputing EMA data, our project is forward‐looking and provides the prospective for various future extensions.

## Conflicts of Interest

The authors declare no conflicts of interest.

## Supporting information


**Data S1.** Supporting Information.

## Data Availability

The MBC1 data can be made available by sending a manuscript proposal and a data use agreement to Bonnie Spring.

## Appendix A Multiple Imputation Sampling for Three Models

In Section 2, we present the general two‐step Bayesian approach used for our multiple imputation. In this appendix, we provide more specifics of each step, tailored to the different settings of the three models.

### RILM

In the first step, sample L parameter sets ${\left\{{\hat{\beta }}_{0}^{l},{\hat{\beta }}^{l},{\hat{\alpha }}_{0}^{l},{\left\{{\hat{v}}_{0,i}^{l}\right\}}_{i=1,\ldots ,N}\right\}}_{l=1,\ldots ,L}$ from the posterior distribution of parameters conditional on the observed data, which is $P\left(\beta_{0},\beta ,\alpha_{0},{\left\{v_{0,i}\right\}}_{i=1,\ldots ,N}|y_{obs}\right)$, and calculate

(A1)
$$\begin{array}{ll} & {\hat{\mu }}_{ij}^{l}={\hat{\beta }}_{0}^{l}+x_{ij}^{⊤}{\hat{\beta }}^{l}+{\hat{v}}_{0,i}^{l} \end{array}$$

(A2)
$$\begin{array}{ll} & {\hat{\sigma_{\epsilon }^{2}}}^{l}=e^{{\hat{\alpha }}_{0}^{l}}. \end{array}$$

In the second step, sample

(A3)
$$\begin{array}{ll} & {ŷ}_{ij}^{l}∼𝒩\left({\hat{\mu }}_{ij}^{l},{\hat{\sigma_{\epsilon }^{2}}}^{l}\right). \end{array}$$

By repeating sampling L times for each parameter set, we get L imputation values ${\left\{{ŷ}_{ij}^{l}\right\}}_{l=1,\ldots ,L}$.

### MELS

In the first step, sample L parameter sets ${\left\{{\hat{\beta }}_{0}^{l},{\hat{\beta }}^{l},{\hat{\alpha }}_{0}^{l},{\hat{\alpha }}^{l},{\left\{{\hat{v}}_{0,i}^{l}\right\}}_{i=1,\ldots ,N},{\left\{{\hat{v}}_{1,i}^{l}\right\}}_{i=1,\ldots ,N}\right\}}_{l=1,\ldots ,L}$ from the posterior distribution of parameters conditional on observed data, which is $P\left(\beta_{0},\beta ,\alpha_{0},\alpha ,{\left\{v_{0,i}\right\}}_{i=1,\ldots ,N},{\left\{v_{1,i}\right\}}_{i=1,\ldots ,N}|y_{obs}\right)$, and calculate

(A4)
$$\begin{array}{ll} & {\hat{\mu }}_{ij}^{l}={\hat{\beta }}_{0}^{l}+x_{ij}^{⊤}{\hat{\beta }}^{l}+{\hat{v}}_{0,i}^{l} \end{array}$$

(A5)
$$\begin{array}{ll} & {\hat{\sigma_{\epsilon_{ij}}^{2}}}^{l}=e^{{\hat{\alpha }}_{0}^{l}+x_{ij}^{⊤}{\hat{\alpha }}^{l}+{\hat{v}}_{1,i}^{l}}. \end{array}$$

In the second step, sample

(A6)
$$\begin{array}{ll} & {ŷ}_{ij}^{l}∼𝒩\left({\hat{\mu }}_{ij}^{l},{\hat{\sigma_{\epsilon_{ij}}^{2}}}^{l}\right). \end{array}$$

By repeating sampling L times for each parameter set, we get L imputation values ${\left\{{ŷ}_{ij}^{l}\right\}}_{l=1,\ldots ,L}$.

### SPMELS

In the first step, sample L parameter sets ${\left\{{\hat{\beta }}_{0}^{l},{\hat{\beta }}^{l},{\hat{\alpha }}_{0}^{l},{\hat{\alpha }}^{l},{\hat{\gamma }}^{l},{\hat{\delta }}^{l},{\left\{{\hat{\eta }}_{0,i}^{l}\right\}}_{i=1,\ldots ,N},{\left\{{\hat{\eta }}_{1,i}^{l}\right\}}_{i=1,\ldots ,N},{\left\{{\hat{\lambda }}_{i}^{l}\right\}}_{i=1,\ldots ,N}\right\}}_{l=1,\ldots ,L}$ from the posterior distribution of parameters conditional on observed data, which is $P\left(\beta_{0},\beta ,\alpha_{0},\alpha ,\gamma ,\delta ,{\left\{\eta_{0,i}\right\}}_{i=1,\ldots ,N},{\left\{\eta_{1,i}\right\}}_{i=1,\ldots ,N},{\left\{\lambda_{i}\right\}}_{i=1,\ldots ,N}|y_{obs}\right)$, and calculate

(A7)
$$\begin{array}{ll} & {\hat{\mu }}_{ij}^{l}={\hat{\beta }}_{0}^{l}+x_{ij}^{⊤}{\hat{\beta }}^{l}+{\hat{\eta }}_{0,i}^{l}+{\hat{\gamma }}^{l}{\hat{\lambda }}_{i}^{l} \end{array}$$

(A8)
$$\begin{array}{ll} & {\hat{\sigma_{\epsilon_{ij}}^{2}}}^{l}=e^{{\hat{\alpha }}_{0}^{l}+x_{ij}^{⊤}{\hat{\alpha }}^{l}+{\hat{\eta }}_{1,i}^{l}+{\hat{\delta }}^{l}{\hat{\lambda }}_{i}^{l}}. \end{array}$$

In the second step, sample

(A9)
$$\begin{array}{ll} & {ŷ}_{ij}^{l}∼𝒩\left({\hat{\mu }}_{ij}^{l},{\hat{\sigma_{\epsilon_{ij}}^{2}}}^{l}\right). \end{array}$$

By repeating sampling L times for each parameter set, we get L imputation values ${\left\{{ŷ}_{ij}^{l}\right\}}_{l=1,\ldots ,L}$.

## Appendix B Bayesian Estimation for Three Models

### RILM

As a reminder, the RILM model is:

(B1)
$$\begin{array}{ll} & y_{ij}|x_{ij},v_{0,i}∼𝒩\left(\beta_{0}+x_{ij}^{⊤}\beta +v_{0,i},\;\sigma_{\epsilon }^{2}\right) \end{array}$$

(B2)
$$\begin{array}{ll} & v_{0,i}∼𝒩\left(0,\sigma_{v_{0}}^{2}\right) \end{array}$$

(B3)
$$\begin{array}{ll} & \sigma_{\epsilon }^{2}=e^{\alpha_{0}}. \end{array}$$

We can rewrite $v_{0,i}$ as

(B4)
$$\begin{array}{ll} & v_{0,i}=\sigma_{v_{0}}z_{0,i} \end{array}$$

(B5)
$$\begin{array}{ll} & z_{0,i}∼𝒩\left(0,1\right). \end{array}$$

To match the notation in the MELS and SPMELS models, we write $\sigma_{v_{0}}$ here as $s_{11}$. Thus, the set of parameters we want to estimate is:

(B6)
$$\begin{array}{ll} & \theta =\left(\beta_{0},\beta ,\alpha_{0},s_{11},{\left\{z_{0,i}\right\}}_{i=1,\ldots ,N}\right). \end{array}$$

In the RILM model, $y_{obs}$ is the non‐missing response $y_{ij}$. Thus, the likelihood $L(y_{obs}|\theta )$ can be explicitly formulated based on the normal distribution model configuration of RILM.

### MELS

As a reminder, the MELS model is:

(B7)
$$\begin{array}{ll} & y_{ij}|x_{ij},v_{0,i},v_{1,i}∼𝒩\left(\beta_{0}+x_{ij}^{⊤}\beta +v_{0,i},\;\sigma_{\epsilon_{ij}}^{2}\right) \end{array}$$

(B8)
$$\begin{array}{ll} & \left(\begin{array}{c} v_{0,i}\\ v_{1,i} \end{array}\right)∼𝒩\left(\left(\begin{array}{c} 0\\ 0 \end{array}\right),\left(\begin{array}{cc} \sigma_{v_{0}}^{2} & \rho_{v_{0},v_{1}}\sigma_{v_{0}}\sigma_{v_{1}}\\ \rho_{v_{0},v_{1}}\sigma_{v_{0}}\sigma_{v_{1}} & \sigma_{v_{1}}^{2} \end{array}\right)\right) \end{array}$$

(B9)
$$\begin{array}{ll} & \sigma_{\epsilon_{ij}}^{2}=e^{\alpha_{0}+x_{ij}^{⊤}\alpha +v_{1,i}}. \end{array}$$

First, we adopt the Cholesky factorization to decompose the covariance matrix and estimate the variance and correlation of the random effects indirectly. By the Cholesky factorization, we can decompose the covariance matrix, which is a positive‐definite matrix, as the multiplication of a lower‐diagonal matrix and its transpose:

(B10)
$$\begin{array}{ll} & \left(\begin{array}{c} v_{0,i}\\ v_{1,i} \end{array}\right)=\left(\begin{array}{cc} s_{11} & 0\\ s_{21} & s_{22} \end{array}\right)\left(\begin{array}{c} z_{0,i}\\ z_{1,i} \end{array}\right), \end{array}$$

where

(B11)
$$\begin{array}{ll} & s_{11}=\sigma_{v_{0}} \end{array}$$

(B12)
$$\begin{array}{ll} & s_{21}=\sigma_{v_{1}}\rho_{v_{0},v_{1}} \end{array}$$

(B13)
$$\begin{array}{ll} & s_{22}=\sqrt{\sigma_{v_{1}}^{2}-{\left(\sigma_{v_{1}}\rho_{v_{0},v_{1}}\right)}^{2}} \end{array}$$

(B14)
$$\begin{array}{ll} & \left(\begin{array}{c} z_{1,i}\\ z_{2,i} \end{array}\right)∼𝒩\left(0_{2},I_{2}\right). \end{array}$$

After the decomposition, the set of parameters we want to estimate is

(B15)
$$\begin{array}{ll} & \theta =\left(\beta_{0},\beta ,\alpha_{0},\alpha ,s_{11},s_{21},s_{22},{\left\{z_{0,i}\right\}}_{i=1,\ldots ,N},{\left\{z_{1,i}\right\}}_{i=1,\ldots ,N}\right). \end{array}$$

In the MELS model, $y_{obs}$ is the non‐missing response $y_{ij}$. Thus, the likelihood $L(y_{obs}|\theta )$ can be explicitly formulated based on the normal distribution model configuration of MELS.

### SPMELS

As a reminder, the SPMELS model is:

(B16)
$$\begin{array}{ll} & y_{ij}|x_{ij},\eta_{0,i},\eta_{1,i},\lambda_{i}∼𝒩\left(\beta_{0}+x_{ij}^{⊤}\beta +\eta_{0,i}+\gamma \lambda_{i},\sigma_{\epsilon_{ij}}^{2}\right) \end{array}$$

(B17)
$$\begin{array}{ll} & m_{ij}|t_{ij},\lambda_{i}∼ℬ\left(L\left(\tau_{0}+t_{ij}^{⊤}\tau +\lambda_{i}\right)\right) \end{array}$$

(B18)
$$\begin{array}{ll} & \left(\begin{array}{c} \eta_{0,i}\\ \eta_{1,i}\\ \lambda_{i} \end{array}\right)∼𝒩\left(\left(\begin{array}{c} 0\\ 0\\ 0 \end{array}\right),\left(\begin{array}{ccc} \sigma_{\eta_{0}}^{2} & \rho_{\eta_{0},\eta_{1}}\sigma_{\eta_{0}}\sigma_{\eta_{1}} & 0\\ \rho_{\eta_{0},\eta_{1}}\sigma_{\eta_{0}}\sigma_{\eta_{1}} & \sigma_{\eta_{1}}^{2} & 0\\ 0 & 0 & \sigma_{\lambda }^{2} \end{array}\right)\right) \end{array}$$

(B19)
$$\begin{array}{ll} & \sigma_{\epsilon_{ij}}^{2}=e^{\alpha_{0}+x_{ij}^{⊤}\alpha +\eta_{1,i}+\delta \lambda_{i}}. \end{array}$$

First, we adopt the Cholesky factorization to decompose the covariance matrix and estimate the variance and correlation of the random effects indirectly. By the Cholesky factorization, we can decompose the covariance matrix, which is a positive‐definite matrix, as the multiplication of a lower‐diagonal matrix and its transpose:

(B20)
$$\begin{array}{l} \left(\begin{array}{c} \eta_{0,i}\\ \eta_{1,i}\\ \lambda_{i} \end{array}\right)=\left(\begin{array}{ccc} s_{11} & 0 & 0\\ s_{21} & s_{22} & 0\\ 0 & 0 & s_{33} \end{array}\right)\;\left(\begin{array}{c} z_{0,i}\\ z_{1,i}\\ z_{2,i} \end{array}\right), \end{array}$$

where

(B21)
$$\begin{array}{ll} & s_{11}=\sigma_{\eta_{0}} \end{array}$$

(B22)
$$\begin{array}{ll} & s_{21}=\sigma_{\eta_{1}}\rho_{\eta_{0},\eta_{1}} \end{array}$$

(B23)
$$\begin{array}{ll} & s_{22}=\sqrt{\sigma_{\eta_{1}}^{2}-{\left(\sigma_{\eta_{1}}\rho_{\eta_{0},\eta_{1}}\right)}^{2}} \end{array}$$

(B24)
$$\begin{array}{ll} & s_{33}=\sigma_{\lambda } \end{array}$$

(B25)
$$\begin{array}{ll} & \left(\begin{array}{c} z_{0,i}\\ z_{1,i}\\ z_{2,i} \end{array}\right)∼𝒩\left(0_{3},I_{3}\right). \end{array}$$

After the decomposition, the set of parameters we want to estimate is

(B26)
$$\begin{array}{ll} & \theta_{1}=\left(\beta_{0},\beta ,\alpha_{0},\alpha ,\tau_{0},\tau ,\gamma ,\delta ,s_{11},s_{21},s_{22},s_{33},{\left\{z_{0,i}\right\}}_{i=1,\ldots ,N},\right.\\ & \;\left.{\left\{z_{1,i}\right\}}_{i=1,\ldots ,N},{\left\{z_{2,i}\right\}}_{i=1,\ldots ,N}\right). \end{array}$$

Denote $m_{ij}$ as the indicator of whether the observation is missing. In the SPMELS model, $y_{obs}$ contains both the non‐missing response $y_{ij}$ and indicator $m_{ij}$. Since the outcomes of $y_{ij}$ and $m_{ij}$ are independent, we can write the likelihood for subject i, occasion j as $L\left(y_{ij}|\theta \right)L\left(m_{ij}|\theta \right)$. Each $L\left(y_{ij}|\theta \right)$ and $L\left(m_{ij}|\theta \right)$ can be explicitly formulated based on the normal distribution and Bernoulli distribution model configurations of the SPMELS model.

## Appendix C Tables

**TABLE C1:** Bias and coverage rates with $\alpha_{0}^{\left(2\right)}=0.00$ and $\rho_{\eta_{0}^{\left(2\right)},\eta_{1}^{\left(2\right)}}=-0.20$ (or $\gamma^{\left(2\right)}=-0.50$ and $\delta^{\left(2\right)}=0.50$).^a^

Parameter	Value	RILM			MELS			SPMELS		
		Estimate	Bias	Coverage	Estimate	Bias	Coverage	Estimate	Bias	Coverage
$\beta_{0}^{\left(2\right)}$	−2.20	−2.58	−0.38	93	−1.99	0.21	98	−1.75	0.45	96
$\beta_{1}^{\left(2\right)}$	2.00	1.98	−0.02	88	1.99	−0.01	95	2.00	−0.00	97
$\beta_{2}^{\left(2\right)}$	0.02	0.04	0.02	93	0.01	−0.01	98	−0.01	−0.03	96
$\alpha_{0}^{\left(2\right)}$	0.00	−0.81	−0.81	12	0.22	0.22	100	0.08	0.08	100
$\alpha_{1}^{\left(2\right)}$	0.30	—	—	—	0.35	0.05	92	0.32	0.02	97
$\alpha_{2}^{\left(2\right)}$	−0.10	—	—	—	−0.11	−0.01	100	−0.10	−0.00	100
$\gamma^{\left(2\right)}$	−0.50	—	—	—	—	—	—	−0.74	−0.24	90
$\delta^{\left(2\right)}$	0.50	—	—	—	—	—	—	0.68	0.18	90
$\sigma_{v_{0}^{\left(2\right)}}$	1.12	1.08	−0.04	91	1.19	0.07	96	1.26	0.14	94
$\sigma_{v_{1}^{\left(2\right)}}$	1.12	—	—	—	1.17	0.05	98	1.25	0.13	97
$\rho_{v_{0}^{\left(2\right)},v_{1}^{\left(2\right)}}$	−0.36	—	—	—	−0.29	0.07	94	−0.30	0.06	96
$\sigma_{\eta_{0}^{\left(2\right)}}$	1.00	—	—	—	—	—	—	1.02	0.02	96
$\sigma_{\eta_{1}^{\left(2\right)}}$	1.00	—	—	—	—	—	—	1.00	−0.00	93
$\rho_{\eta_{0}^{\left(2\right)},\eta_{1}^{\left(2\right)}}$	−0.20	—	—	—	—	—	—	−0.11	0.09	96
$\sigma_{\lambda }$	1.00	—	—	—	—	—	—	1.25	0.25	95
$\tau_{1}$	0.10	—	—	—	—	—	—	0.11	0.01	91
$\tau_{2}$	0.05	—	—	—	—	—	—	−0.00	−0.05	95
$\tau_{3}$	0.10	—	—	—	—	—	—	0.10	0.00	99

^a^ In Section 3.1, we initially set $\alpha^{\left(2\right)}=0.00$ and $\rho_{\eta_{0},\eta_{1}}^{\left(2\right)}=-0.20$, and these parameters are varied in subsequent simulations. Concurrently, the values of $\gamma^{\left(2\right)}$ and $\delta^{\left(2\right)}$ are consistently maintained at −0.50 and 0.50, respectively. These latter values align with the initial settings for $\gamma^{\left(2\right)}$ and $\delta^{\left(2\right)}$ in Section 3.2. In Section 3.2, we initially set $\gamma^{\left(2\right)}=-0.50$ and $\delta^{\left(2\right)}=0.50$, and these parameters are varied in subsequent simulations. Concurrently, the values of $\alpha^{\left(2\right)}$ and $\rho_{\eta_{0}^{\left(2\right)},\eta_{1}^{\left(2\right)}}$ are consistently maintained at 0.00 and −0.20, respectively. As a result, the table serves a dual purpose: it represents the parameter combination of $\alpha^{\left(2\right)}=0.00$, $\rho_{\eta_{0}^{\left(2\right)},\eta_{1}^{\left(2\right)}}=-0.20$ from Section 3.1 and the combination $\gamma^{\left(2\right)}=-0.50$, $\delta^{\left(2\right)}=0.50$ from Section 3.2.

**TABLE C2:** Bias and coverage rates with $\alpha_{0}^{\left(2\right)}=3.00$ and $\rho_{\eta_{0}^{\left(2\right)},\eta_{1}^{\left(2\right)}}=-0.20$.

Parameter	Value	RILM			MELS			SPMELS		
		Estimate	Bias	Coverage	Estimate	Bias	Coverage	Estimate	Bias	Coverage
$\beta_{0}^{\left(2\right)}$	−2.20	−2.91	−0.71	91	−1.77	0.43	99	−1.59	0.61	97
$\beta_{1}^{\left(2\right)}$	2.00	1.91	−0.09	78	1.97	−0.03	96	2.01	0.01	94
$\beta_{2}^{\left(2\right)}$	0.02	0.06	0.04	87	0.00	−0.02	100	−0.01	−0.03	97
$\alpha_{0}^{\left(2\right)}$	3.00	2.15	−0.85	4	1.33	−1.67	43	1.23	−1.77	31
$\alpha_{1}^{\left(2\right)}$	0.30	—	—	—	0.31	0.01	94	0.28	−0.02	95
$\alpha_{2}^{\left(2\right)}$	−0.10	—	—	—	−0.02	0.08	52	−0.01	0.09	43
$\gamma^{\left(2\right)}$	−0.50	—	—	—	—	—	—	−0.71	−0.21	94
$\delta^{\left(2\right)}$	0.50	—	—	—	—	—	—	0.79	0.29	94
$\sigma_{v_{0}^{\left(2\right)}}$	1.12	1.07	−0.05	89	1.19	0.07	95	1.27	0.15	94
$\sigma_{v_{1}^{\left(2\right)}}$	1.12	—	—	—	1.35	0.23	85	1.42	0.31	81
$\rho_{v_{0}^{\left(2\right)},v_{1}^{\left(2\right)}}$	−0.36	—	—	—	−0.28	0.08	97	−0.31	0.05	99
$\sigma_{\eta_{0}^{\left(2\right)}}$	1.00	—	—	—	—	—	—	0.99	−0.01	97
$\sigma_{\eta_{1}^{\left(2\right)}}$	1.00	—	—	—	—	—	—	1.16	0.16	93
$\rho_{\eta_{0}^{\left(2\right)},\eta_{1}^{\left(2\right)}}$	−0.20	—	—	—	—	—	—	−0.12	0.08	95
$\sigma_{\lambda_{i}}$	1.00	—	—	—	—	—	—	0.88	−0.12	95
$\tau_{1}$	0.10	—	—	—	—	—	—	0.11	0.01	94
$\tau_{2}$	0.05	—	—	—	—	—	—	0.00	−0.05	94
$\tau_{3}$	0.10	—	—	—	—	—	—	0.10	0.00	99

**TABLE C3:** Imputation errors with different $\alpha_{0}^{\left(2\right)}$ (the intercept of the within‐variance) and $\rho_{\eta_{0}^{\left(2\right)},\eta_{1}^{\left(2\right)}}$ (the correlation between residual random location and random scale effects).

Method to impute $x_{1}$		RILM								
Method to impute y		RILM		MELS		SPMELS		Best^a^	Worst^b^	Difference ratio (%)^c^
Imputation method		Single	Multiple	Single	Multiple	Single	Multiple			
$\alpha_{0}^{\left(2\right)}$	$\rho_{\eta_{0}^{\left(2\right)},\eta_{1}^{\left(2\right)}}$									
0.00	−0.20	8.53	6.52	6.23	5.56	6.29	5.57	MELS	RILM	14.72
1.00	−0.20	11.46	8.51	9.44	7.42	9.48	7.41	SPMELS	RILM	12.93
2.00	−0.20	17.84	11.87	15.93	10.78	16.17	10.83	MELS	RILM	9.18
3.00	−0.20	37.84	24.77	36.58	22.25	36.34	22.26	MELS	RILM	10.17
0.00	−0.80	9.18	6.68	6.49	5.75	6.49	5.75	MELS	RILM	13.92

Method to impute $x_{1}$		MELS								
Method to impute y		RILM		MELS		SPMELS		Best	Worst	Difference ratio (%)
Imputation method		Single	Multiple	Single	Multiple	Single	Multiple			
$\alpha_{0}^{\left(2\right)}$	$\rho_{\eta_{0}^{\left(2\right)},\eta_{1}^{\left(2\right)}}$									
0.00	−0.20	7.31	5.15	5.11	4.46	5.13	4.47	MELS	RILM	13.40
1.00	−0.20	9.93	7.05	8.18	6.20	8.20	6.23	MELS	RILM	12.06
2.00	−0.20	17.55	10.92	14.87	9.74	14.74	9.73	SPMELS	RILM	10.90
3.00	−0.20	35.78	23.31	34.77	21.03	34.66	20.99	SPMELS	RILM	9.95
0.00	−0.80	8.06	5.41	5.33	4.63	5.34	4.64	MELS	RILM	14.42

Method to impute $x_{1}$		SPMELS								
Method to impute y		RILM		MELS		SPMELS		Best	Worst	Difference ratio (%)
Imputation method		Single	Multiple	Single	Multiple	Single	Multiple			
$\alpha_{0}^{\left(2\right)}$	$\rho_{\eta_{0}^{\left(2\right)},\eta_{1}^{\left(2\right)}}$									
0.00	−0.20	7.21	5.04	5.04	4.37^d^	5.05	4.37^d^	SPMELS	RILM	13.29
1.00	−0.20	9.86	6.94	8.07	6.10	8.08	6.09	SPMELS	RILM	12.25
2.00	−0.20	17.58	10.82	14.60	9.60	14.78	9.63	MELS	RILM	11.28
3.00	−0.20	35.66	23.29	35.05	20.91	34.65	20.98	MELS	RILM	10.22
0.00	−0.80	7.97	5.29	5.22	4.54	5.23	4.53	SPMELS	RILM	14.37

^a^ The best model refers to the model with the smallest error.

^b^ The worst model refers to the model with the largest error.

^c^ The difference ratio represents the percentage difference between the model's largest and smallest errors, with the difference normalized by the largest error. For example, the difference ratio “14.72” is calculated by $100\%\times \frac{6.52-5.56}{6.52}\approx 14.72\%$.

^d^ The values displayed in the table have been rounded to two decimal places. These two values look the same, but before rounding, the error of SPMELS is smaller than MELS. Thus, SPMELS is the best model.

**TABLE C4:** Bias and coverage rates with $\alpha_{0}^{\left(2\right)}=0.00$ and $\rho_{\eta_{0}^{\left(2\right)},\eta_{1}^{\left(2\right)}}=-0.80$.

Parameter	Value	RILM			MELS			SPMELS		
		Estimate	Bias	Coverage	Estimate	Bias	Coverage	Estimate	Bias	Coverage
$\beta_{0}^{\left(2\right)}$	−2.20	−2.85	−0.65	92	−2.23	−0.03	100	−2.05	0.15	99
$\beta_{1}^{\left(2\right)}$	2.00	1.99	−0.01	81	2.00	−0.00	95	2.00	0.00	95
$\beta_{2}^{\left(2\right)}$	0.02	0.05	0.03	93	0.02	−0.00	100	0.01	−0.01	97
$\alpha_{0}^{\left(2\right)}$	0.00	−0.80	−0.80	14	0.13	0.13	100	−0.03	−0.03	99
$\alpha_{1}^{\left(2\right)}$	0.30	—	—	—	0.33	0.03	94	0.31	0.01	94
$\alpha_{2}^{\left(2\right)}$	−0.10	—	—	—	−0.11	−0.01	100	−0.09	0.01	100
$\gamma^{\left(2\right)}$	−0.50	—	—	—	—	—	—	−0.78	−0.28	88
$\delta^{\left(2\right)}$	0.50	—	—	—	—	—	—	0.76	0.26	91
$\sigma_{v_{0}^{\left(2\right)}}$	1.12	1.07	−0.05	94	1.14	0.02	97	1.20	0.08	96
$\sigma_{v_{1}^{\left(2\right)}}$	1.12	—	—	—	1.12	0.00	94	1.19	0.07	93
$\rho_{v_{0}^{\left(2\right)},v_{1}^{\left(2\right)}}$	−0.84	—	—	—	−0.78	0.06	99	−0.78	0.06	99
$\sigma_{\eta_{0}^{\left(2\right)}}$	1.00	—	—	—	—	—	—	0.93	−0.07	92
$\sigma_{\eta_{1}^{\left(2\right)}}$	1.00	—	—	—	—	—	—	0.92	−0.08	93
$\rho_{\eta_{0}^{\left(2\right)},\eta_{1}^{\left(2\right)}}$	−0.80	—	—	—	—	—	—	−0.66	0.14	99
$\sigma_{\lambda }$	1.00	—	—	—	—	—	—	1.19	0.19	95
$\tau_{1}$	0.10	—	—	—	—	—	—	0.11	0.01	93
$\tau_{2}$	0.05	—	—	—	—	—	—	0.00	−0.05	95
$\tau_{3}$	0.10	—	—	—	—	—	—	0.10	0.00	99

**TABLE C5:** Bias and coverage rates with $\gamma^{\left(2\right)}=-1.50$ and $\delta^{\left(2\right)}=0.50$.

Parameter	Value	RILM			MELS			SPMELS		
		Estimate	Bias	Coverage	Estimate	Bias	Coverage	Estimate	Bias	Coverage
$\beta_{0}^{\left(2\right)}$	−2.20	−3.59	−1.39	96	−2.27	−0.07	100	−1.96	0.24	99
$\beta_{1}^{\left(2\right)}$	2.00	1.99	−0.01	86	1.99	−0.01	94	2.00	−0.00	96
$\beta_{2}^{\left(2\right)}$	0.02	0.08	0.06	97	0.01	−0.01	100	−0.00	−0.02	96
$\alpha_{0}^{\left(2\right)}$	0.00	−0.81	−0.81	14	0.07	0.07	100	0.00	0.00	100
$\alpha_{1}^{\left(2\right)}$	0.30	—	—	—	0.33	0.03	97	0.32	0.02	97
$\alpha_{2}^{\left(2\right)}$	−0.10	—	—	—	−0.10	−0.00	100	−0.10	0.00	100
$\gamma^{\left(2\right)}$	−1.50	—	—	—	—	—	—	−1.90	−0.40	90
$\delta^{\left(2\right)}$	0.50	—	—	—	—	—	—	0.54	0.04	96
$\sigma_{v_{0}^{\left(2\right)}}$	1.80	1.69	−0.11	95	1.87	0.06	99	1.90	0.10	99
$\sigma_{v_{1}^{\left(2\right)}}$	1.12	—	—	—	1.17	0.05	97	1.24	0.12	96
$\rho_{v_{0}^{\left(2\right)},v_{1}^{\left(2\right)}}$	−0.47	—	—	—	−0.37	0.11	96	−0.38	0.09	97
$\sigma_{\eta_{0}^{\left(2\right)}}$	1.00	—	—	—	—	—	—	0.95	−0.05	97
$\sigma_{\eta_{1}^{\left(2\right)}}$	1.00	—	—	—	—	—	—	1.05	0.05	95
$\rho_{\eta_{0}^{\left(2\right)},\eta_{1}^{\left(2\right)}}$	−0.20	—	—	—	—	—	—	−0.13	0.07	99
$\sigma_{\lambda_{i}}$	1.00	—	—	—	—	—	—	1.24	0.24	97
$\tau_{1}$	0.10	—	—	—	—	—	—	0.11	0.01	93
$\tau_{2}$	0.05	—	—	—	—	—	—	0.00	−0.05	94
$\tau_{3}$	0.10	—	—	—	—	—	—	0.10	−0.00	99

**TABLE C6:** Bias and coverage rates with $\gamma^{\left(2\right)}=-0.50$ and $\delta^{\left(2\right)}=1.00$.

Parameter	Value	RILM			MELS			SPMELS		
		Estimate	Bias	Coverage	Estimate	Bias	Coverage	Estimate	Bias	Coverage
$\beta_{0}^{\left(2\right)}$	−2.20	−2.74	−0.54	96	−2.04	0.16	98	−1.92	0.28	95
$\beta_{1}^{\left(2\right)}$	2.00	1.98	−0.02	80	2.00	0.00	94	2.00	0.00	90
$\beta_{2}^{\left(2\right)}$	0.02	0.04	0.02	94	0.01	−0.01	97	0.00	−0.02	94
$\alpha_{0}^{\left(2\right)}$	0.00	−0.39	−0.39	42	0.35	0.35	100	0.12	0.12	100
$\alpha_{1}^{\left(2\right)}$	0.30	—	—	—	0.36	0.06	86	0.32	0.02	93
$\alpha_{2}^{\left(2\right)}$	−0.10	—	—	—	−0.11	−0.01	100	−0.10	0.00	98
$\gamma^{\left(2\right)}$	−0.50	—	—	—	—	—	—	−0.71	−0.21	96
$\delta^{\left(2\right)}$	1.00	—	—	—	—	—	—	1.29	0.29	93
$\sigma_{v_{0}^{\left(2\right)}}$	1.12	1.07	−0.05	92	1.18	0.06	96	1.24	0.12	96
$\sigma_{v_{1}^{\left(2\right)}}$	1.41	—	—	—	1.42	0.00	98	1.49	0.08	99
$\rho_{v_{0}^{\left(2\right)},v_{1}^{\left(2\right)}}$	−0.44	—	—	—	−0.40	0.04	96	−0.41	0.03	95
$\sigma_{\eta_{0}^{\left(2\right)}}$	1.00	—	—	—	—	—	—	1.02	0.02	98
$\sigma_{\eta_{1}^{\left(2\right)}}$	1.00	—	—	—	—	—	—	0.95	−0.05	96
$\rho_{\eta_{0}^{\left(2\right)},\eta_{1}^{\left(2\right)}}$	−0.20	—	—	—	—	—	—	−0.12	0.08	98
$\sigma_{\lambda_{i}}$	1.00	—	—	—	—	—	—	1.49	0.49	95
$\tau_{1}$	0.10	—	—	—	—	—	—	0.11	0.01	92
$\tau_{2}$	0.05	—	—	—	—	—	—	0.00	−0.05	95
$\tau_{3}$	0.10	—	—	—	—	—	—	0.10	0.00	98

**TABLE C7:** Imputation errors with different $\gamma^{\left(2\right)}$ (the effect coefficient of the random missing effect on the mean of y) and $\delta^{\left(2\right)}$ (the effect coefficient of the random missing effect on the variance of y).

Method to impute $x_{1}$		RILM								
Method to impute y		RILM		MELS		SPMELS		Best^a^	Worst^b^	Difference ratio (%)^c^
Imputation method		Single	Multiple	Single	Multiple	Single	Multiple			
$\gamma^{\left(2\right)}$	$\delta^{\left(2\right)}$									
−0.50	0.50	8.53	6.52	6.23	5.56	6.29	5.57	MELS	RILM	14.72
−1.00	0.50	12.14	7.04	6.73	5.91	6.79	5.92	MELS	RILM	16.05
−1.50	0.50	11.00	6.80	6.50	5.76	6.53	5.79	MELS	RILM	15.29
−0.50	0.75	9.46	7.01	6.90	5.97	6.87	6.00	MELS	RILM	14.84
−0.50	1.00	9.81	7.12	7.69	6.36	7.68	6.39	MELS	RILM	10.67

Method to impute $x_{1}$		MELS								
Method to impute y		RILM		MELS		SPMELS		Best	Worst	Difference ratio (%)
Imputation method		Single	Multiple	Single	Multiple	Single	Multiple			
$\gamma^{\left(2\right)}$	$\delta^{\left(2\right)}$									
−0.50	0.50	7.31	5.15	5.11	4.46	5.13	4.47	MELS	RILM	13.40
−1.00	0.50	10.14	5.74	5.56	4.77	5.60	4.79	MELS	RILM	16.90
−1.50	0.50	9.59	5.65	5.29	4.62	5.35	4.62	SPMELS	RILM	18.23
−0.50	0.75	8.05	5.71	5.78	4.90	5.74	4.91	MELS	RILM	14.19
−0.50	1.00	8.52	5.98	6.53	5.29	6.58	5.29	MELS	RILM	11.54

Method to impute $x_{1}$		SPMELS								
Method to impute y		RILM		MELS		SPMELS		Best	Worst	Difference ratio (%)
Imputation method		Single	Multiple	Single	Multiple	Single	Multiple			
$\gamma^{\left(2\right)}$	$\delta^{\left(2\right)}$									
−0.50	0.50	7.21	5.04	5.04	4.37^d^	5.05	4.37^d^	SPMELS	RILM	13.29
−1.00	0.50	10.12	5.69	5.49	4.70	5.52	4.72	MELS	RILM	17.40
−1.50	0.50	9.47	5.54	5.18	4.53	5.27	4.53	MELS	RILM	18.23
−0.50	0.75	7.94	5.56	5.68	4.76	5.57	4.76	MELS	RILM	14.39
−0.50	1.00	8.43	5.88	6.48	5.20	6.48	5.18	SPMELS	RILM	11.90

^a^ The best model refers to the model with the smallest error.

^b^ The worst model refers to the model with the largest error.

^c^ The difference ratio represents the percentage difference between the model's largest and smallest errors, with the difference normalized by the largest error. For example, the difference ratio “14.72” is calculated by $100\%\times \frac{6.52-5.56}{6.52}\approx 14.72\%$.

^d^ The values displayed in the table have been rounded to two decimal places. These two values look the same, but before rounding, the error of SPMELS is smaller than MELS. Thus, SPMELS is the best model.

**TABLE C8:** Demographic and clinical variables of the participants.

Variable	Value
Gender (n,%)	
0: Male	48 (23.76)
1: Female	154 (76.24)
Age (M,SD,range)	32.81 ± 11.02 (21.00‐60.00)
Condition (n,%)	
0: Increase MVPA	93 (46.04)
1: Decrease sedentary minutes	109 (53.96)
Sedentary minutes (M,SD,range)	401.45 ± 240.70 (0.00‐1150.00)
MVPA minutes (M,SD,range)	28.31 ± 28.01 (0.00‐272.00)

**TABLE C9:** Parameter estimates and intervals for sedentary minutes (square root form).

Parameter	RILM		MELS		SPMELS	
	Estimate	95% Credible Interval	Estimate	95% Credible Interval	Estimate	95% Credible Interval
$\beta_{intercept}$	24.32	(23.40, 25.23)	24.33	(23.36, 25.21)	24.57	(23.60, 25.45)
$\beta_{gender}$	−1.12	(−1.75, −0.49)	−1.09	(−1.66, −0.51)	−0.99	(−1.54, −0.39)
$\beta_{age}$	−0.06	(−0.28, 0.16)	−0.07	(−0.30, 0.16)	−0.16	(−0.38, 0.07)
$\beta_{condition}$	0.01	(−0.54, 0.57)	+0.00^a^	(−0.50, 0.51)	−0.12	(−0.62, 0.41)
$\beta_{Rx1}$	−0.08	(−0.37, 0.21)	−0.12	(−0.37, 0.13)	−0.11	(−0.36, 0.14)
$\beta_{Rx23}$	−0.35	(−0.60, −0.11)	−0.37	(−0.59, −0.15)	−0.36	(−0.58, −0.15)
$\beta_{weekend}$	−1.07	(−1.30, −0.83)	−1.10	(−1.33, −0.85)	−1.09	(−1.32, −0.85)
$\beta_{Rx1*condition}$	−0.26	(−0.66, 0.18)	−0.20	(−0.56, 0.18)	−0.22	(−0.57, 0.15)
$\beta_{Rx23*condition}$	0.06	(−0.29, 0.37)	0.07	(−0.23, 0.39)	0.06	(−0.26, 0.38)
$\beta_{Rx1*weekend}$	−0.57	(−1.08, −0.05)	−0.56	(−1.10, −0.04)	−0.59	(−1.12, −0.09)
$\beta_{Rx23*weekend}$	−0.28	(−0.68, 0.14)	−0.31	(−0.75, 0.12)	−0.33	(−0.78, 0.11)
$\beta_{Rx1*condition*weekend}$	0.25	(−0.42, 0.89)	0.36	(−0.31, 1.04)	0.40	(−0.23, 0.99)
$\beta_{Rx23*condition*weekend}$	0.38	(−0.06, 0.86)	0.52	(0.03, 1.00)	0.52	(−0.00, 1.02)
$\alpha_{intercept}$	1.65	(1.61, 1.69)	1.46	(1.19, 1.73)	1.45	(1.17, 1.73)
$\alpha_{gender}$	—	—	0.06	(−0.12, 0.23)	0.04	(−0.12, 0.20)
$\alpha_{age}$	—	—	−0.03	(−0.09, 0.04)	−0.02	(−0.08, 0.05)
$\alpha_{condition}$	—	—	0.23	(0.05, 0.40)	0.24	(0.06, 0.42)
$\alpha_{Rx1}$	—	—	0.04	(−0.15, 0.24)	0.04	(−0.14, 0.21)
$\alpha_{Rx23}$	—	—	0.10	(−0.05, 0.26)	0.10	(−0.06, 0.26)
$\alpha_{weekend}$	—	—	0.18	(0.01, 0.35)	0.18	(0.03, 0.34)
$\alpha_{Rx1*condition}$	—	—	−0.22	(−0.49, 0.04)	−0.22	(−0.48, 0.05)
$\alpha_{Rx23*condition}$	—	—	−0.18	(−0.39, 0.04)	−0.17	(−0.39, 0.05)
$\alpha_{Rx1*weekend}$	—	—	0.19	(−0.16, 0.53)	0.20	(−0.13, 0.54)
$\alpha_{Rx23*weekend}$	—	—	0.25	(−0.02, 0.52)	0.26	(−0.00, 0.52)
$\alpha_{Rx1*condition*weekend}$	—	—	−0.31	(−0.73, 0.12)	−0.32	(−0.73, 0.09)
$\alpha_{Rx23*condition*weekend}$	—	—	−0.23	(−0.52, 0.08)	−0.22	(−0.55, 0.07)
$\tau_{intercept}$	—	—	—	—	−1.12	(−1.35, −0.90)
$\tau_{weekend}$	—	—	—	—	0.68	(0.56, 0.81)
$\tau_{Rx1}$	—	—	—	—	−0.48	(−0.65, −0.31)
$\tau_{Rx23}$	—	—	—	—	−0.04	(−0.17, 0.08)
γ	—	—	—	—	−0.46	(−0.65, −0.26)
δ	—	—	—	—	0.07	(0.01, 0.13)
$\sigma_{v_{0}}$	1.78	(1.59, 1.99)	1.78	(1.60, 1.98)	1.79	(1.59, 1.99)
$\sigma_{v_{1}}$	—	—	0.39	(0.33, 0.46)	0.40	(0.34, 0.46)
$\sigma_{\eta_{0}}$	—	—	—	—	1.66	(1.49, 1.86)
$\sigma_{\eta_{1}}$	—	—	—	—	0.38	(0.33, 0.45)
$\sigma_{\lambda }$	—	—	—	—	1.39	(1.24, 1.56)
$\rho_{v_{0},v_{1}}$	—	—	0.02	(−0.16, 0.21)	−0.01	(−0.18, 0.18)
$\rho_{\eta_{0},\eta_{1}}$	—	—	—	—	0.10	(−0.09, 0.28)
ELPD	−10890.93		−10767.78		−10766.08	

^a^ The values displayed in the table have been rounded to two decimal places. A value of “+0.00” indicates that the original number, before rounding, was greater than 0. Conversely, “‐0.00” signifies that the original number was less than 0 before rounding.

**TABLE C10:** Parameter estimates and intervals for MVPA (square root form).

Parameter	RILM		MELS		SPMELS	
	Estimate	95% Credible Interval	Estimate	95% Credible Interval	Estimate	95% Credible Interval
$\beta_{intercept}$	9.00	(8.10, 9.82)	8.57	(7.76, 9.37)	8.60	(7.74, 9.43)
$\beta_{sedentary\;minutes}$	−0.10	(−0.13, −0.08)	−0.08	(−0.10, −0.06)	−0.08	(−0.10, −0.06)
$\beta_{gender}$	−0.48	(−0.89, −0.08)	−0.47	(−0.90, −0.06)	−0.47	(−0.92, −0.06)
$\beta_{age}$	−0.23	(−0.39, −0.07)	−0.24	(−0.39, −0.07)	−0.25	(−0.42, −0.09)
$\beta_{condition}$	0.26	(−0.14, 0.65)	0.20	(−0.17, 0.57)	0.20	(−0.20, 0.61)
$\beta_{Rx1}$	0.08	(−0.13, 0.30)	0.08	(−0.10, 0.25)	0.08	(−0.09, 0.25)
$\beta_{Rx23}$	−0.09	(−0.28, 0.09)	−0.15	(−0.31, 0.01)	−0.15	(−0.32, 0.02)
$\beta_{weekend}$	−1.14	(−1.33, −0.95)	−1.13	(−1.32, −0.93)	−1.12	(−1.32, −0.93)
$\beta_{Rx1*condition}$	0.59	(0.29, 0.88)	0.61	(0.36, 0.88)	0.61	(0.35, 0.87)
$\beta_{Rx23*condition}$	1.10	(0.84, 1.35)	1.13	(0.90, 1.37)	1.13	(0.89, 1.37)
$\beta_{Rx1*weekend}$	+0.00^a^	(−0.42, 0.40)	−0.00	(−0.39, 0.41)	−0.01	(−0.43, 0.37)
$\beta_{Rx23*weekend}$	0.43	(0.12, 0.75)	0.41	(0.07, 0.75)	0.40	(0.07, 0.73)
$\beta_{Rx1*condition*weekend}$	0.27	(−0.23, 0.80)	0.23	(−0.30, 0.76)	0.23	(−0.30, 0.79)
$\beta_{Rx23*condition*weekend}$	−0.30	(−0.66, 0.06)	−0.24	(−0.64, 0.17)	−0.23	(−0.61, 0.16)
$\alpha_{intercept}$	1.15	(1.11, 1.19)	1.94	(1.39, 2.43)	1.95	(1.42, 2.47)
$\alpha_{sedentary\;minutes}$	—	—	−0.02	(−0.04, −0.00)	−0.02	(−0.04, −0.00)
$\alpha_{gender}$	—	—	−0.12	(−0.30, 0.05)	−0.12	(−0.31, 0.06)
$\alpha_{age}$	—	—	−0.11	(−0.18, −0.04)	−0.11	(−0.18, −0.04)
$\alpha_{condition}$	—	—	−0.07	(−0.27, 0.12)	−0.08	(−0.27, 0.11)
$\alpha_{Rx1}$	—	—	−0.20	(−0.38, −0.01)	−0.20	(−0.38, −0.01)
$\alpha_{Rx23}$	—	—	−0.07	(−0.23, 0.09)	−0.07	(−0.23, 0.10)
$\alpha_{weekend}$	—	—	0.38	(0.22, 0.55)	0.38	(0.22, 0.54)
$\alpha_{Rx1*condition}$	—	—	0.12	(−0.15, 0.39)	0.13	(−0.14,0.39)
$\alpha_{Rx23*condition}$	—	—	0.19	(−0.03, 0.42)	0.19	(−0.03, 0.42)
$\alpha_{Rx1*weekend}$	—	—	0.15	(−0.20, 0.50)	0.14	(−0.19, 0.48)
$\alpha_{Rx23*weekend}$	—	—	0.21	(−0.05, 0.49)	0.21	(−0.05, 0.49)
$\alpha_{Rx1*condition*weekend}$	—	—	0.05	(−0.4, 0.49)	0.04	(−0.38,0.47)
$\alpha_{Rx23*condition*weekend}$	—	—	−0.27	(−0.61, 0.04)	−0.27	(−0.58, 0.06)
$\tau_{intercept}$	—	—	—	—	−1.12	(−1.34, −0.91)
$\tau_{weekend}$	—	—	—	—	0.68	(0.55, 0.81)
$\tau_{Rx1}$	—	—	—	—	−0.48	(−0.65, −0.32)
$\tau_{Rx23}$	—	—	—	—	−0.04	(−0.17, 0.09)
γ	—	—	—	—	−0.03	(−0.18, 0.12)
δ	—	—	—	—	−0.02	(−0.09, 0.04)
$\sigma_{v_{0}}$	1.23	(1.10, 1.37)	1.27	(1.14, 1.41)	1.28	(1.14,1.44)
$\sigma_{v_{1}}$	—	—	0.44	(0.38, 0.51)	0.45	(0.38, 0.52)
$\sigma_{\eta_{0}}$	—	—	—	—	1.27	(1.14, 1.43)
$\sigma_{\eta_{1}}$	—	—	—	—	0.44	(0.38, 0.52)
$\sigma_{\lambda }$	—	—	—	—	1.40	(1.24, 1.57)
$\rho_{v_{0},v_{1}}$	—	—	0.21	(0.03, 0.39)	0.21	(0.01, 0.40)
$\rho_{\eta_{0},\eta_{1}}$	—	—	—	—	0.21	(0.01, 0.40)
ELPD	−9688.50		−9480.24		−9480.31	

^a^ The values displayed in the table have been rounded to two decimal places. A value of “+0.00” indicates that the original number, before rounding, was greater than 0. Conversely, “‐0.00” signifies that the original number was less than 0 before rounding.
